# Peroxisomal fission is modulated by the mitochondrial Rho‐GTPases, Miro1 and Miro2

**DOI:** 10.15252/embr.201949865

**Published:** 2020-01-02

**Authors:** Christian Covill‐Cooke, Viktoriya S Toncheva, James Drew, Nicol Birsa, Guillermo López‐Doménech, Josef T Kittler

**Affiliations:** ^1^ Neuroscience, Physiology and Pharmacology Department University College London London UK

**Keywords:** Fis1, oscillatory, Rhot1, Rhot2, tail‐anchored, Membrane & Intracellular Transport

## Abstract

Peroxisomes are essential for a number of cellular functions, including reactive oxygen species metabolism, fatty acid β‐oxidation and lipid synthesis. To ensure optimal functionality, peroxisomal size, shape and number must be dynamically maintained; however, many aspects of how this is regulated remain poorly characterised. Here, we show that the localisation of Miro1 and Miro2—outer mitochondrial membrane proteins essential for mitochondrial trafficking—to peroxisomes is not required for basal peroxisomal distribution and long‐range trafficking, but rather for the maintenance of peroxisomal size and morphology through peroxisomal fission. Mechanistically, this is achieved by Miro negatively regulating Drp1‐dependent fission, a function that is shared with the mitochondria. We further find that the peroxisomal localisation of Miro is regulated by its first GTPase domain and is mediated by an interaction through its transmembrane domain with the peroxisomal‐membrane protein chaperone, Pex19. Our work highlights a shared regulatory role of Miro in maintaining the morphology of both peroxisomes and mitochondria, supporting a crosstalk between peroxisomal and mitochondrial biology.

## Graphical abstract

Miro1 and Miro2 localise to peroxisomes and negatively regulate peroxisomal fission rather than long‐range transport and distribution.

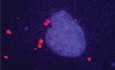

## Introduction

Peroxisomes are single membrane‐bound organelles that are required for a wide range of essential metabolic pathways. As sites of both the production and clearance of reactive oxygen species (ROS) and the synthesis of a subset of lipids (e.g. plasmalogens), peroxisomes are critical for cellular health. The importance of peroxisomes is emphasised by loss‐of‐function mutations of key genes in peroxisomal biogenesis (*PEX* genes) leading to Zellweger spectrum disorders 1. As peroxisomes have a role in metabolism, they are known to respond to environmental cues by altering their size, number and distribution to ensure optimal functionality 2.

Peroxisomal morphology and size can be rapidly altered through fission of pre‐existing, mature peroxisomes. Peroxisomal fission also serves as an important mechanism of peroxisomal biogenesis alongside *de novo* formation, the combination of pre‐peroxisomal vesicles from the endoplasmic reticulum and mitochondria 3, 4, 5. To initiate peroxisomal fission, the peroxisome must first elongate through the membrane curving properties of Pex11β 6, 7. Following elongation, peroxisomal fission can occur at several sites leading to the formation of multiple peroxisomes from the initial mature seed. Strikingly, the latter steps of peroxisomal fission require overlapping machinery with mitochondrial fission, with Fis1 and Mff being localised to peroxisomes for the recruitment of the GTPase Drp1 from the cytoplasm 8, 9, 10. Once recruited to the peroxisomal membrane, Drp1 is proposed to oligomerise, which leads to sufficient force to sever the peroxisomal membrane 11. Analogy with mitochondrial fission is often made; however, the extent of the overlap in mechanism and whether there are shared regulatory processes between the peroxisomes and mitochondria are poorly understood.

As peroxisomes are involved in a diverse range of metabolic functions and the fact that they interact with several organelles 12, peroxisomes must also be trafficked throughout the cell. The importance of this has been emphasised by *SPAST* mutant cells exhibiting reduced peroxisomal trafficking and, subsequently, defects in distribution, which results in impaired handling of ROS 13, 14. The current paradigm of peroxisomal trafficking in mammalian cells is that ~10% of peroxisomes undergo long‐range microtubule‐dependent trafficking using kinesin‐1 and dynein, with the rest exhibiting shorter‐range displacements 15, 16, 17, 18, 19, 20. Despite the importance of peroxisomal dynamics, the mechanisms that regulate trafficking and distribution are not well defined.

An increasing number of proteins are now known to be shared between mitochondria and peroxisomes with roles in many aspects of organelle homeostasis. These include Fis1, Mff, Drp1, GDAP1, USP30, MUL1/MAPL, OMP25, MAVS, BCL‐XL, BCL‐2 and more recently Miro1/2 6, 10, 21, 22, 23, 24, 25, 26. The mitochondrial Rho‐GTPases, Miro1 and Miro2, are outer mitochondrial membrane (OMM) proteins critical for mitochondrial trafficking 27, 28, 29, 30, 31. Structurally, both Miro paralogues exhibit a large, cytoplasm‐facing N‐terminus with two calcium‐binding EF‐hand domains flanked by a GTPase domain on each side 32, 33. Here, we confirm that Miro1 and Miro2 are not strictly localised to mitochondria but are also localised to peroxisomes. Moreover, the peroxisomal localisation of Miro is regulated through its first GTPase domain and requires the transmembrane domain for binding with the cytosolic chaperone, Pex19. Taking advantage of Miro knockout mouse embryonic fibroblasts (MEFs), we find that in contrast to previous reports, and its role at mitochondria, Miro is not required to establish steady‐state peroxisomal distribution through long‐range microtubule‐dependent trafficking 26, 31, 34. Instead, we show that the Miro family of proteins modulate peroxisomal morphology and size by negatively regulating Drp1‐dependent fission. As a result, we propose an overarching role for Miro in the coordination and maintenance of peroxisomal and mitochondrial size and shape.

## Results

**Figure 1:**
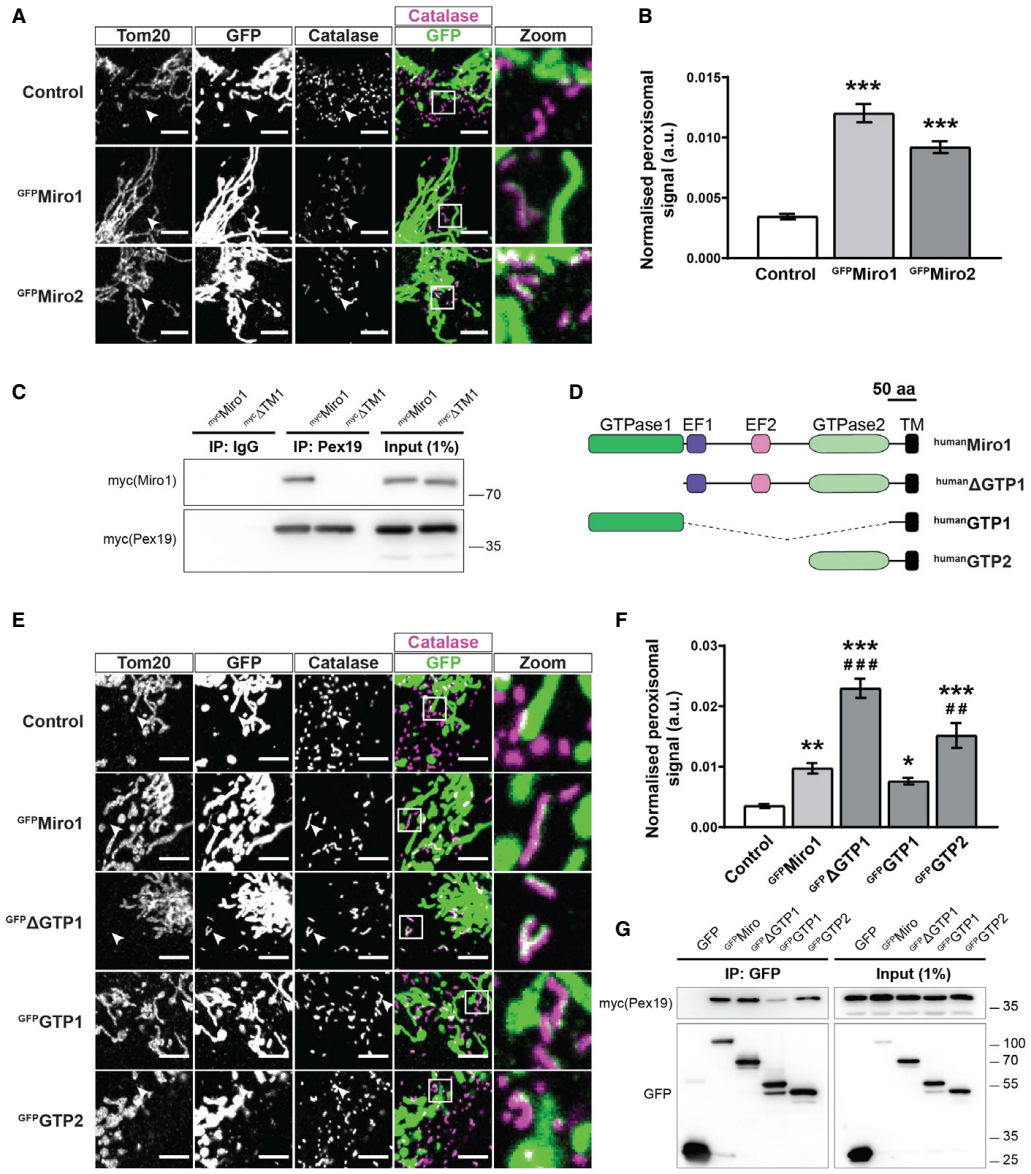
The first GTPase domain and transmembrane domain of Miro1 control its peroxisomal localisation Representative zooms of WT MEFs transfected with GFP‐tagged Miro1 and Miro2 (^GFP^Miro1 and ^GFP^Miro2). Control is GFP fused to the first 70 amino acids of Tom70. Tom20 and catalase stain mitochondria and peroxisomes, respectively. Merge is of Miro (green) and catalase (magenta) with co‐localisation shown as white. White arrowheads highlight an area where an isolated peroxisome is situated.Quantification of the extent of GFP signal on peroxisomes.Pulldown of overexpressed Pex19 in Cos7 cells with a Pex19 antibody shows interaction with full‐length Miro1 (^myc^Miro1), but not Miro1 lacking the transmembrane domain (^myc^ΔTM1).Schematic of Miro1 truncation mutants used in E‐G. TM denotes the transmembrane domain.Representative images of Miro1 truncation constructs expressed in DKO MEFs. Mitochondria and peroxisomes are stained with Tom20 and catalase, respectively. White arrowheads highlight an area where an isolated peroxisome is situated.Quantification of the extent of peroxisomal localisation of the Miro1 truncation constructs.Co‐immunoprecipation of Miro1 truncation constructs and ^myc^Pex19 following transfection into Cos7 cells. GFP‐tagged Miro1 truncation constructs were pulled down with GFP‐Trap agarose beads, and Pex19 was probed with myc antibody.Data information: (B and F) One‐way ANOVA with Newman–Keuls *post hoc* test was used for all comparisons (*n* = 18 cells per condition over three independent experiments). *, ** and *** denote *P* < 0.05, *P* < 0.01 and *P* < 0.001 in comparison with control, respectively, and ^##^ and ^###^ denote *P* < 0.01 and *P* < 0.001 in comparison with ^GFP^Miro1, respectively. Data are represented as mean ± SEM. Scale bar is 5 μm.

Recent reports have shown that Miro1 and Miro2 can localise to peroxisomes 24, 26, 34. To confirm these results and to develop a quantitative assay for measuring changes in the localisation of Miro, GFP‐tagged human Miro1 (^GFP^Miro1) and Miro2 (^GFP^Miro2) were expressed in MEFs. Alongside their well‐documented mitochondrial localisation 32, 35, both Miro1 and Miro2 were found to localise with peroxisomes, as seen by co‐localisation with catalase staining (Fig 1A). To measure the extent of this peroxisomal localisation, GFP signal on catalase (peroxisomes) positive but Tom20 (mitochondria) negative structures was quantified (see Materials and Methods). Both ^GFP^Miro1 and ^GFP^Miro2 showed a significant enrichment in peroxisomal localisation over control (a GFP fusion protein of the mitochondria‐targeting sequence of Tom70 (amino acids 1–70)), highlighting a specific localisation of Miro to the peroxisomes and not simply a mislocalisation of OMM proteins (Fig 1A and B).

The targeting of proteins to the peroxisomal membrane has been shown to require the cytosolic chaperone, Pex19 36, 37, 38. Like Miro1/2, Fis1 is dually targeted to both mitochondria and peroxisomes, a process dependent on the C‐terminus (transmembrane domain and adjacent amino acids) of Fis1 binding to Pex19 39. Indeed, other peroxisomal‐membrane proteins have been shown to bind to Pex19 via their C‐termini 37, 40. As a result, the importance of the transmembrane domain of Miro1 to bind Pex19 was explored. To achieve this, we expressed ^myc^Pex19 and both full‐length ^myc^Miro1 and ^myc^Miro1ΔTM (Miro1 lacking its transmembrane domain) in Cos7 cells. By pulling down Pex19, with an anti‐Pex19 antibody, we observed robust co‐immunoprecipitation of Miro1 with Pex19 (Fig 1C). Interestingly, this interaction was completely abolished upon deletion of the transmembrane domain. Therefore, both Miro1 and Miro2 can localise to peroxisomes and the Miro transmembrane domain is critical for its interaction with Pex19.

Being anchored in the OMM by their C‐termini, both Miro1 and Miro2 exhibit a large cytoplasm‐facing N‐terminus. Structurally, the N‐terminal part of the proteins includes two EF‐hand domains flanked by a GTPase domain on either side. To characterise the importance of these domains in the peroxisomal localisation of Miro, we generated truncation constructs of Miro1 (Fig 1D) and expressed them in Miro1/Miro2 double knockout (DKO) MEFs to prevent the influence of any endogenous Miro. Strikingly, Miro1 constructs lacking the first GTPase domain (^GFP^ΔGTP1 and ^GFP^GTP2) exhibited a dramatic increase in peroxisomal localisation in comparison with full‐length Miro1 (Fig 1E and F). This was particularly exaggerated in the case of ^GFP^ΔGTP1. In contrast, ^GFP^GTP1 showed no peroxisomal enrichment compared to ^GFP^Miro1. Given that the loss of the first GTPase domain enhanced the localisation of Miro1 to peroxisomes, we tested whether it influenced the binding of Miro1 to Pex19. Cos7 cells were transfected with both the ^GFP^Miro1 truncation constructs (Fig 1G) and ^myc^Pex19. Following pulldown of GFP, ^myc^Pex19 was found to co‐immunoprecipitate with the full‐length ^GFP^Miro1, ^GFP^ΔGTP1 and ^GFP^GTP2 forms of Miro1 (Fig 1G). Interestingly, binding of ^myc^Pex19 and ^GFP^GTP1 occurred to a much lesser extent, suggesting that the first GTPase domain may negatively regulate the ability of Pex19 to bind to the transmembrane domain of Miro1. This observation, along with the enhanced peroxisomal localisation of ^GFP^ΔGTP1 (lacking GTPase domain 1; Fig 1E and F), suggests that GTPase domain 1 plays an important regulatory role in the peroxisomal targeting of Miro1.

**Figure EV1:**
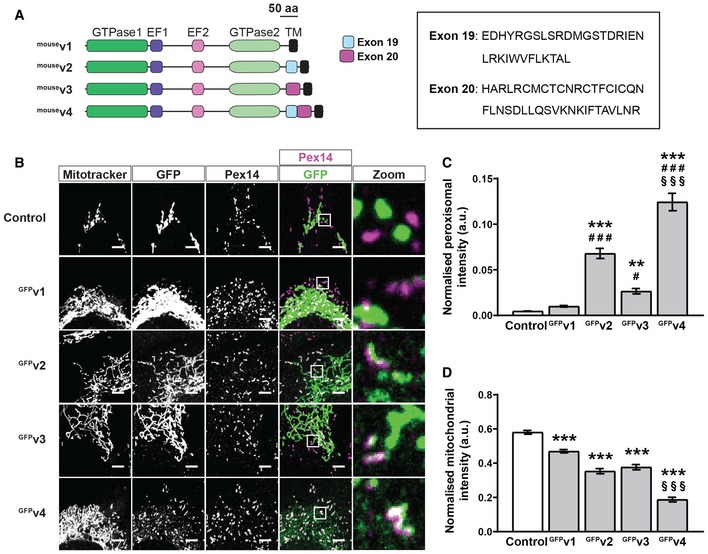
Localisation of mouse Miro1 splice variants in DKOMEFs Schematic of mouse Miro1 splice variants including amino acid sequences of exon 19 and exon 20. TM denotes the transmembrane domain.Representative images of DKO MEFs expressing control (GFP‐tagged 1‐70 of Tom70) or mouse Miro1 splice variants (^GFP^v1, ^GFP^v2, ^GFP^v3 and ^GFP^v4 corresponding to variants 1, 2, 3 and 4, respectively). MitoTracker stains mitochondria, and Pex14 stains peroxisomes. Scale bar is 5 μm.Comparison of Miro1 splice variant localisation to peroxisomes by thresholded GFP signal on Pex14‐positive and MitoTracker‐negative structures.Comparison of splice variant localisation to mitochondria by thresholded GFP signal on MitoTracker‐positive and Pex14‐negative structures.Data information: For (C) and (D), *n* = 30 cells per condition over three independent experiments. ** denotes *P* < 0.01 in comparison with control. ^#^ is *P* < 0.05 compared to ^GFP^v1. ***, ^###^ and ^§§§^ denote *P* < 0.001 in comparison with control, ^GFP^v1 and ^GFP^v2, respectively, by one‐way ANOVA with Newman–Keuls *post hoc* test. Data are represented as mean ± SEM.

**Figure EV2:**
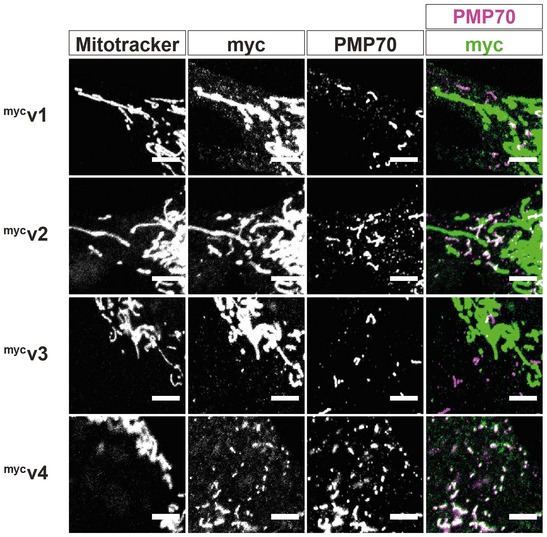
Characterisation of myc‐tagged mouse Miro1 splice variants Representative images of myc‐tagged mouse Miro1 splice variants (^myc^v1, ^myc^v2, ^myc^v3 and ^myc^v4 corresponding to variants 1, 2, 3 and 4, respectively). MitoTracker stains mitochondria, and PMP70 stains peroxisomes. Scale bar is 5 μm.

Interestingly, human splice variants of Miro1 have been identified (variant 2, variant 3 and variant 4, including exon 19, 20 and both 19 and 20, respectively) and shown to have differential localisation to mitochondria and peroxisomes 26. More specifically, the inclusion of exon 19 (found in variant 2 and variant 4) was shown to promote localisation to peroxisomes. We expressed the mouse Miro1 splice variants—variant 1: ^GFP^v1; variant 2: ^GFP^v2; variant 3: ^GFP^v3; and variant 4: ^GFP^v4 (Fig EV1A)—in the DKO MEFs to prevent the competition with endogenous Miro1 or Miro2 and quantified their levels at mitochondria and peroxisomes. Exon 19 and exon 20 in mice are 84% and 95% identical to exon 19 and exon 20 in humans, respectively. By co‐staining mitochondria and peroxisomes, we found that—as reported in Okumoto *et al* 26 for the human variants—mouse ^GFP^v4 exhibited a predominantly peroxisomal localisation, whereas ^GFP^v2 exhibited a shared mitochondrial and peroxisomal localisation (Fig EV1B–D). In contrast to Okumoto *et al* 26, however, we found that ^GFP^v3 also showed a higher peroxisomal localisation over ^GFP^v1, highlighting that exon 20 can also promote the peroxisomal localisation of Miro1. Myc‐tagged mouse variants showed similar staining as the GFP‐tagged versions (Fig EV2), further confirming the effects of exon 19 and exon 20 on the peroxisomal localisation of Miro1. Therefore, the first GTPase domain, exon 19, exon 20 and the transmembrane domain are all key features in modulating the peroxisomal localisation of Miro1.

**Figure 2:**
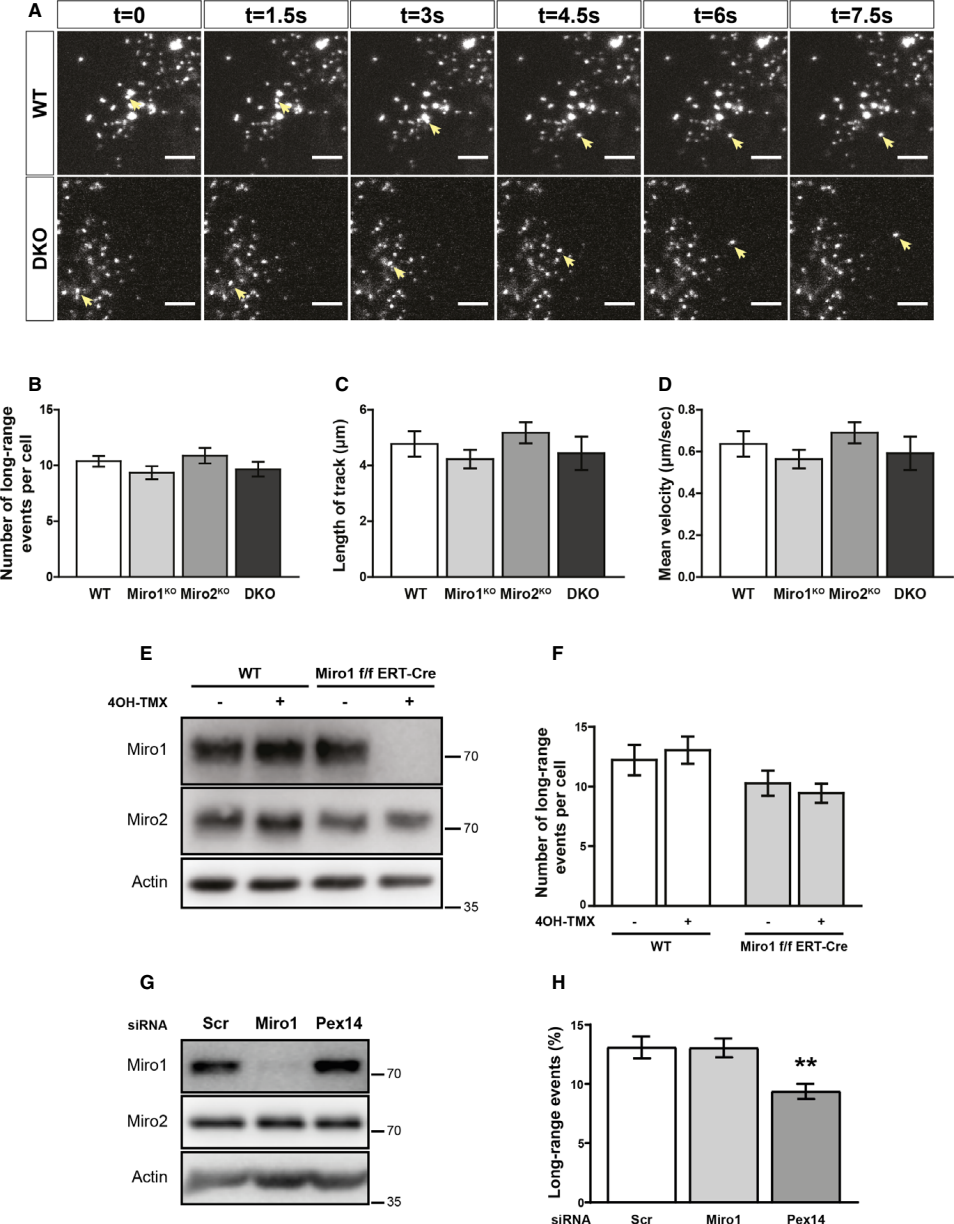
Loss of Miro1 or Miro2 does not affect long‐range peroxisomal trafficking ASix consecutive frames of live trafficking of peroxisomes (pxDsRed signal at 1.5 s per frame). Yellow arrows show the trajectory of a fast‐moving peroxisome in both WT and DKO MEFs. Scale bar is 5 μm.BBlind scoring of the number of long‐range peroxisomal trafficking events in WT, Miro1^KO^ and Miro2^KO^ and DKO MEFs (*n* = 42 cells over six independent experiments. Two different MEFs lines were used for each genotype).C, DQuantification of individual track length and velocity, respectively (*n* = 24 cells over three independent experiments).ERepresentative blots of Miro1, Miro2 and actin from whole cell lysates of wild‐type and Miro1‐floxed ERT‐Cre‐recombinase MEFs treated with and without 4‐OH tamoxifen for 48 hours. Lysates were taken 1 day after the end of treatment.FQuantification of long‐range peroxisomal trafficking events by blind scoring of pxDsRed signal from a two‐minute movie (*n* = 18 cells per condition over three independent experiments).GRepresentative blot of Miro1 and Miro2 following knockdown of either Miro1 or Pex14 in HeLa cells.HQuantification of long‐range peroxisomal trafficking events (visualised with pxGFP) in HeLa cells transfected with scrambled, Miro1 and Pex14 siRNA, by blind scoring of a 5‐min movie (*n* = 18 cells over three independent experiments).Data information: For (B) a Kruskal–Wallis with a Dunn's correction *post hoc* test was used to test for significance. Analysis for (C), (D) and (H) is a one‐way ANOVA with Newman–Keuls *post hoc* test. ** denotes *P* < 0.01. Statistical significance in (F) was calculated by two‐way ANOVA. For (B), (C), (D), (F) and (H), no statistical difference between conditions was observed, unless stated. Data are represented as mean ± SEM.

Following the identification of key features critical for the peroxisomal localisation of Miro, we next sought to better understand the function of Miro at peroxisomes. Miro has been extensively documented to be critical for mitochondrial distribution through bidirectional microtubule‐dependent trafficking in a wide variety of species and cell types 31, 41, 42, 43, 44. Peroxisomes also dynamically maintain their distribution through microtubule‐dependent trafficking events with long‐range peroxisomal trafficking accounting for ~10% of peroxisomal transport 15, 16, 17, 19, 45, 46, 47. Recently, it has been proposed that Miro1 can regulate long‐range peroxisomal trafficking 26, 34. Given this, and the overlap in the mechanism of long‐range trafficking between mitochondria and peroxisomes, a role for Miro in basal microtubule‐dependent peroxisomal transport and distribution was explored. To observe the Miro dependency of long‐range peroxisomal trafficking, pxDsRed (DsRed2 localised to peroxisomes by a peroxisomal lumen targeting signal, PTS1) was transfected into wild‐type (WT), Miro1 single knockout (Miro1^KO^), Miro2 single knockout (Miro2^KO^) and DKO MEFs and imaged at one frame every 1.5 s for 2 min (Movie EV1, EV2, EV3 and EV4). Long‐range peroxisomal trafficking events were then quantified by blind scoring (see Materials and Methods). Surprisingly, in contrast to the role of Miro in mitochondrial transport, quantification of the number of long‐range peroxisomal trafficking events showed no difference in this behaviour between WT, Miro1^KO^, Miro2^KO^ and DKO MEFs (Fig 2A and B; [Link], [Link]). Depolymerisation of microtubules by vinblastine abolished long‐ranged trafficking, as reported previously, highlighting that the trafficking events quantified were in fact microtubule‐dependent (Fig EV4C; [Link], [Link]) 15, 16, 17, 19, 20, 45, 48. To investigate whether the properties of the trafficking events themselves were altered upon the loss of Miro, individual long‐ranged trajectories were manually tracked. Quantification of run‐length and mean velocity showed no difference between WT, Miro1^KO^, Miro2^KO^ and DKO MEFs (Fig 2C and D), further confirming that the loss of Miro does not affect basal long‐range trafficking.

To account for any long‐term compensatory mechanisms resulting from the chronic loss of Miro, we next tested whether the acute loss of Miro1 reduced peroxisomal transport. To induce the acute loss of Miro1, we treated Miro1‐floxed MEFs expressing a tamoxifen‐inducible Cre‐recombinase (Miro1^f/f^ ERT‐Cre) with 4‐OH tamoxifen for 48 h and imaged pxDsRed following a further day in culture. Using this treatment paradigm, Miro1 is undetectable by Western blot, whilst Miro2 levels remained comparable to untreated MEFs (Fig 2E). Comparison of wild‐type and Miro1^f/f^ ERT‐Cre MEFs both with and without 4‐OH tamoxifen treatment showed no significant difference in long‐range peroxisomal trafficking events (Fig 2F). To further test the effect of acute loss of Miro1 on peroxisomal transport, we knocked down Miro1 in HeLa cells using a previously characterised siRNA 26. A dramatic loss of Miro1, but not Miro2, protein levels was observed 48 h after transfection (Fig 2G). In agreement with the live imaging in MEFs, a reduction in Miro1 protein did not cause a decrease in long‐range peroxisomal transport (Fig 2H), whereas a significant decrease in long‐range peroxisomal transport was observed following knockdown of Pex14, in agreement with previous work 20. Therefore, both the acute loss of Miro1 and the chronic loss of Miro1/2 do not significantly impact microtubule‐dependent peroxisomal transport.

**Figure EV3:**
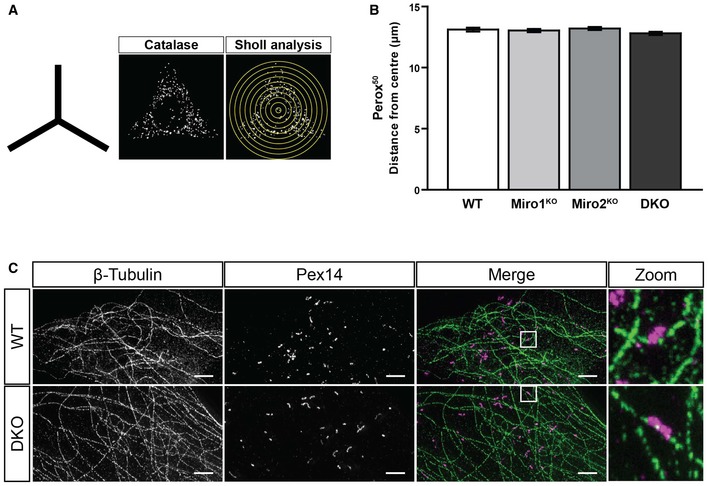
Peroxisomes can still associate with microtubules following the loss of Miro Schematic of the shape of the fibronectin patterns used for organelle distribution experiments and representative image of catalase signal of WT MEF on a micropattern along with a schematic of Sholl analysis.Quantification of the distance at which 50% of peroxisomal signal is situated in WT, Miro1^KO^, Miro2^KO^ and DKO MEFs following Sholl analysis (*n* = 60 cells per condition over three independent experiments). No statistical significance was observed following a one‐way ANOVA. All data are represented as mean ± SEM.Representative images of STED imaging of β‐tubulin (microtubules in green) and Pex14 (peroxisomes in magenta) in WT and DKO MEFs. Scale bar is 2 μm.

**Figure 3:**
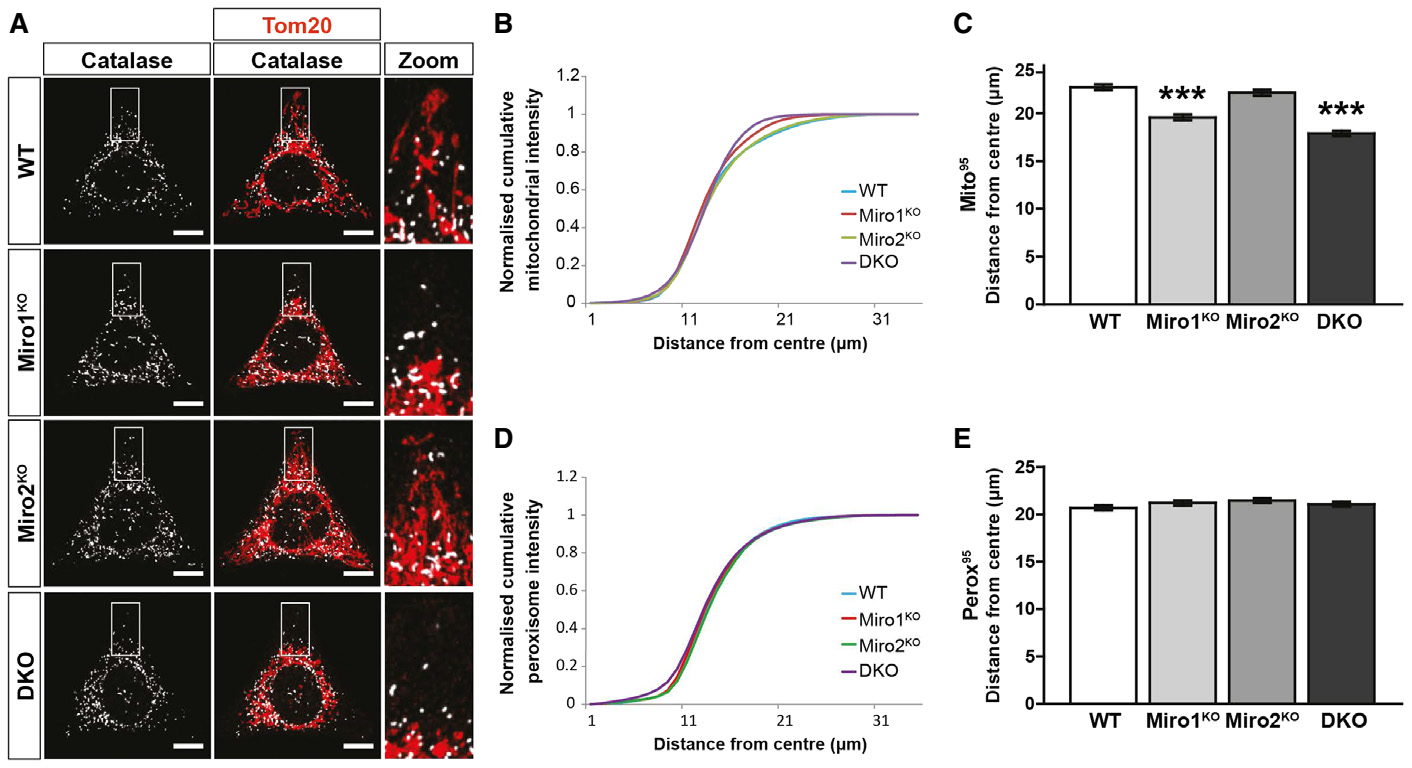
Loss of Miro does not impact basal peroxisomal distribution Representative images of WT, Miro1^KO^, Miro2^KO^ and DKO MEFs seeded onto Y‐shaped fibronectin micropatterns stained for mitochondria (Tom20 in red) and peroxisomes (catalase in white). Scale bar represents 10 μm.Normalised cumulative distribution of mitochondria in WT, Miro1^KO^, Miro2^KO^ and DKO MEFs.Distance at which 95% of mitochondria are distributed (Mito^95^) for all four genotypes of MEF.Normalised cumulative distribution curves comparing the peroxisomal distribution from the centre of the cell to the periphery of WT, Miro1^KO^, Miro2^KO^ and DKO MEFs.Bar graph comparing the average distance at which 95% of the peroxisomal signal is distributed (Perox^95^).Data information: (A–E) is *n* = 60 cells over three independent experiments. For (C) and (E), a one‐way ANOVA with Newman–Keuls *post hoc* test was used to test for statistical significance. *** represents *P* < 0.001. Data are represented as mean ± SEM.

In accordance with the requirement of Miro for long‐range microtubule‐dependent mitochondrial transport, the loss of Miro also dramatically affects the positioning of mitochondria, with mitochondria becoming more perinuclear in distribution 30, 31, 44, 49, 50. To quantify whether Miro is also required to establish peroxisomal distribution, a Sholl‐based quantification method was applied 30, 31. Briefly, WT, Miro1^KO^, Miro2^KO^ and DKO MEFs were seeded on Y‐shaped fibronectin micropatterns to standardise cell morphology, then fixed and stained for a peroxisomal and mitochondrial marker. Organelle distribution was then measured by concentric circles being drawn from the centre of the cell at 1‐μm intervals and the inter‐circle organelle marker signal being quantified, and plotted with distance (Fig EV3A) 31. As expected, mitochondrial distribution was shifted significantly towards the nucleus in both Miro1^KO^ and DKO MEFs in comparison with WT cells, whereas the loss of Miro2 had no effect (Fig 3A–C) 31. When quantifying peroxisomal distribution between the four genotypes of MEFs, no difference was observed in the normalised cumulative distribution (Fig 3D). Quantification of the distance at which 50% (Perox^50^) and 95% (Perox^95^) of peroxisomes are situated further supports this conclusion, with no differences between any genotype being observed (Figs 3A and E, and EV3B). To test whether the localisation of peroxisomes on the microtubule network was altered upon the loss of Miro, we carried out two‐colour STED super‐resolution microscopy of peroxisomes (labelled with Pex14) and microtubules (labelled with β‐tubulin) in the WT and DKO MEFs (Fig EV3C). By imaging at ~40 nm resolution, peroxisomes were observed to associate extensively with microtubules in both WT and DKO MEFs (Fig EV3C). Altogether, in contrast to the established role of Miro in microtubule‐dependent mitochondrial trafficking and distribution, Miro does not have a role in maintaining steady‐state peroxisomal distribution through long‐range peroxisomal transport.

**Figure 4:**
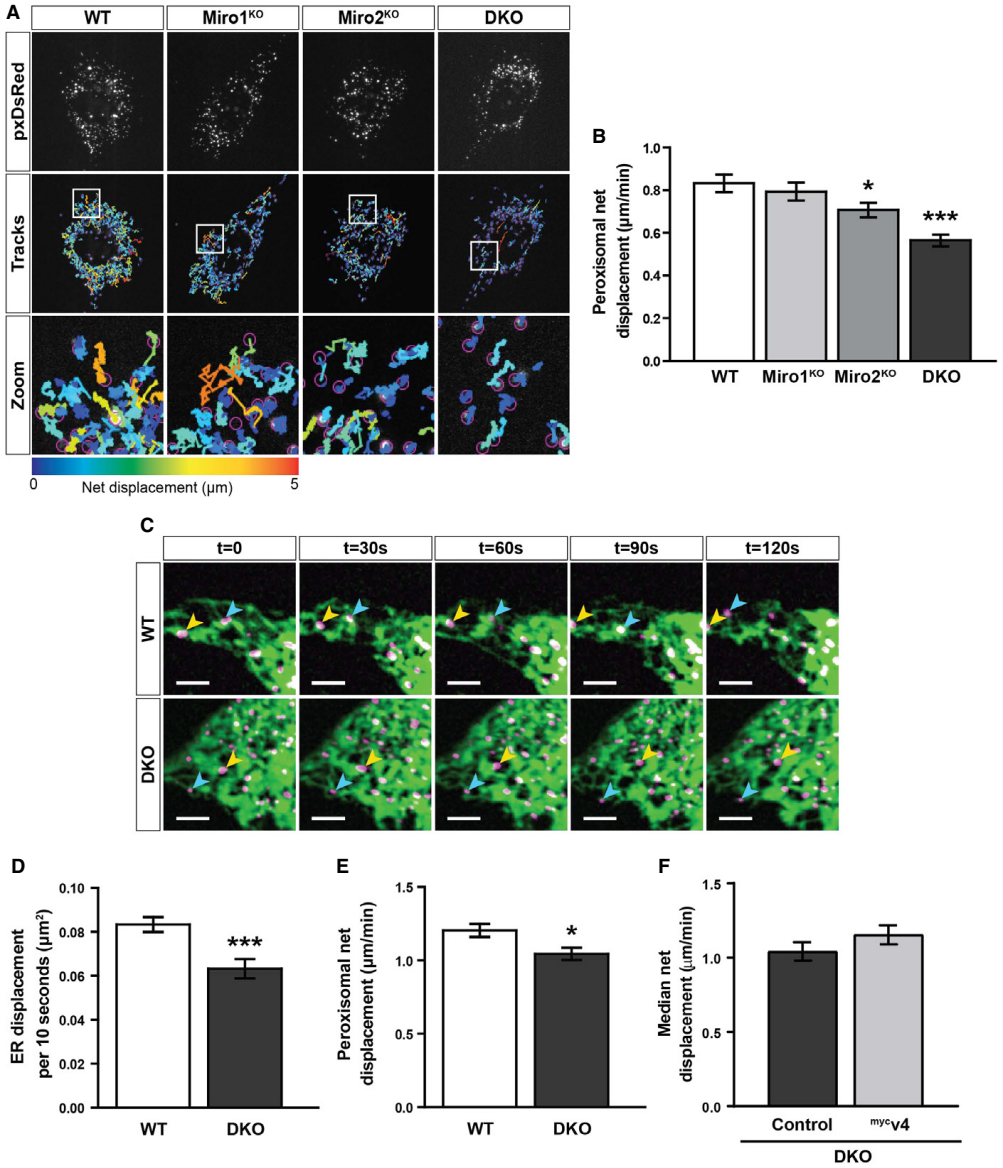
Loss of Miro1/2 reduces short‐range peroxisomal displacements Snapshots and tracks of pxDsRed signal in WT, Miro1^KO^, Miro2^KO^ and DKO MEFs following live imaging at 1.5 s per frame for 2 min.Quantification of median net displacement of individual peroxisomes (pxDsRed signal) for WT, Miro1^KO^, Miro2^KO^ and DKO MEFs (*n* = 42 cells over six independent experiments. Two different MEFs lines were used for each genotype).Still images every 30 s of ER and peroxisomes (ER‐DsRed pseudo‐coloured to green and pxGFP pseudo‐coloured to magenta, respectively) in WT and DKO MEFs taken from 2‐min movies by live‐cell spinning‐disc microscopy. Arrows track individual peroxisomes associated with an ER tubule. Scale bar is 5 μm.Quantification of the relative ER displacement over a 2‐min movie using ER‐DsRed signal in WT and DKO MEFs (*n* = 20 cells per condition over three independent experiments).Median net displacement of peroxisomes in WT and DKO MEFs. *n* = 20 cells per condition over three independent experiments.Median net displacement of peroxisomes in DKO MEFs with and without myc‐tagged variant 4 of Miro1 (^myc^v4) (*n* = 12 cells per condition over three independent experiments).Data information: For multiple comparisons in (B), a one‐way ANOVA with Newman–Keuls *post hoc* test was used to test for statistical significance. For (D–F), Student's *t*‐test was used to calculate statistical significance. * and *** represent *P* < 0.05 and *P* < 0.001, respectively. Data are represented as mean ± SEM.

**Figure EV4:**
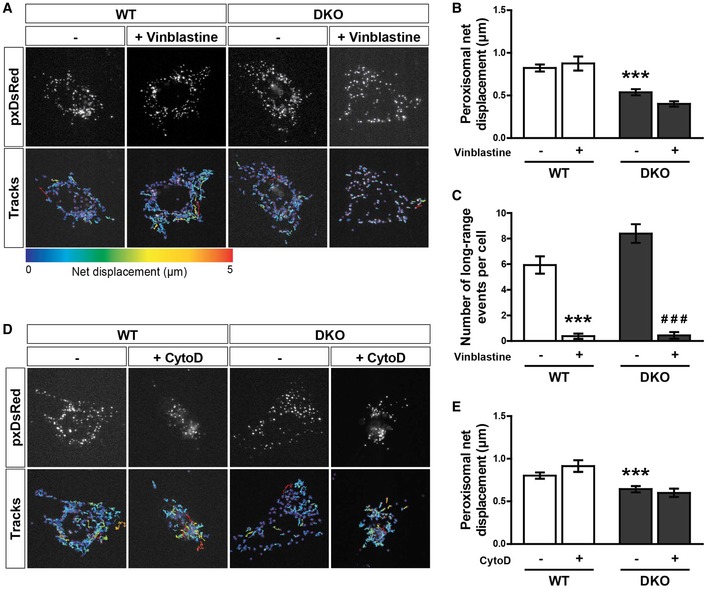
The effect of perturbation to the microtubules and actin on peroxisomal motility Snapshots and tracks from representative movies of peroxisome motility (pxDsRed) of untreated and vinblastine‐treated (1 μM for 1 h) WT and DKO MEFs. Movies were acquired at 1.5 s per frame for 2 min.Median net displacement of peroxisomes over a 2‐min period (*n* = 16 cells per condition over three independent experiments).Blind scoring of long‐ranged peroxisomal trafficking events per cell over a 2‐min movie. *n* = 16 cells per condition over three independent experiments.Snapshots and tracks from representative movies of peroxisome motility (pxDsRed) of untreated and cytochalasin‐D (CytoD)‐treated (1 μg/ml for 30 min) WT and DKO MEFs. Movies were acquired at 1.5 s per frame for 2 min.Median net displacement of peroxisomes from untreated and cytochalasin‐D‐treated WT and DKO MEFs (*n* = 18 cells per condition over three independent experiments).Data information: Two‐way ANOVA was used to test for significance; *** and ^###^ denote *P* < 0.001 in comparison with WT untreated and DKO untreated MEFs, respectively. Data are represented as mean ± SEM.

Approximately 90% of peroxisomal trafficking occurs by shorter‐range displacements. In contrast to long‐range peroxisomal transport, the mechanism by which shorter‐range trafficking is regulated is not well defined 15, 17, 20. As we did not find Miro to be required for long‐range peroxisomal trafficking and distribution, we tested whether it has a role in shorter‐range peroxisomal displacements. To quantify this, individual peroxisomes were automatically tracked in WT, Miro1^KO^, Miro2^KO^ and DKO MEFs over time. Interestingly, we observed a significant reduction in the median net displacement of peroxisomes in both Miro2^KO^ and, to a greater extent, DKO MEFs in comparison with WT cells (Fig 4A and B). No difference was observed between WT and Miro1^KO^ MEFs. To probe into the role of Miro in this type of trafficking, we first tested the importance of the microtubule and actin cytoskeletons in WT and DKO MEFs. Depolymerisation of microtubules or actin—by vinblastine or cytochalasin‐D, respectively—in WT and DKO MEFs had no effect on median net displacement of peroxisomes (Fig EV4A–E; Movie EV7, EV8, EV9 and EV10), in agreement with previous work 15, 16, 51. As a result, Miro appears to regulate short‐range peroxisomal transport; however, this is unlikely modulated by either the actin or microtubule cytoskeletons.

Miro2 has recently been found to localise to the ER 24. Given this, and the fact that Miro2^KO^ cells show a reduction in short‐range trafficking, we next sought to investigate the relationship of the ER and short‐range peroxisomal trafficking. In fact, it has been long known that peroxisomes make extensive contact with the ER 52. WT and DKO MEFs were transfected with pxGFP and ER‐DsRed and co‐imaged by live spinning‐disc microscopy. In WT cells, peroxisomes were observed to associate significantly with the ER. Interestingly, this association occurred throughout the movie with the peroxisomes apparently following the path of the ER (Fig 4C; Movie EV11). Indeed, the ER has been shown to oscillate 53. As the shorter‐range displacements of peroxisomes appeared to follow the oscillation of the ER, Miro could be regulating the oscillations of the ER or the association of peroxisomes with the ER. Dual‐organelle imaging in the DKO MEFs still showed significant association of peroxisomes with the ER (Fig 4C; Movie EV12); however, when quantifying ER displacement with time, a significant reduction in ER oscillation was observed in the DKO MEFs in comparison with WT cells (Fig 4D). Therefore, it is possible that the reduction in shorter‐range peroxisomal displacements in the Miro2^KO^ and DKO MEFs is a result of defects in ER trafficking (Fig 4E). To test whether rescuing peroxisomal Miro1 could increase shorter‐range peroxisomal transport following the loss of Miro, ^myc^v4 was transfected into DKO MEFs. No significant increase in peroxisomal transport was observed between control DKO MEFs and those transfected with ^myc^v4 (Fig 4F). Consequently, Miro mainly appears to be important for shorter‐range peroxisomal displacements, likely through the ER.

**Figure 5:**
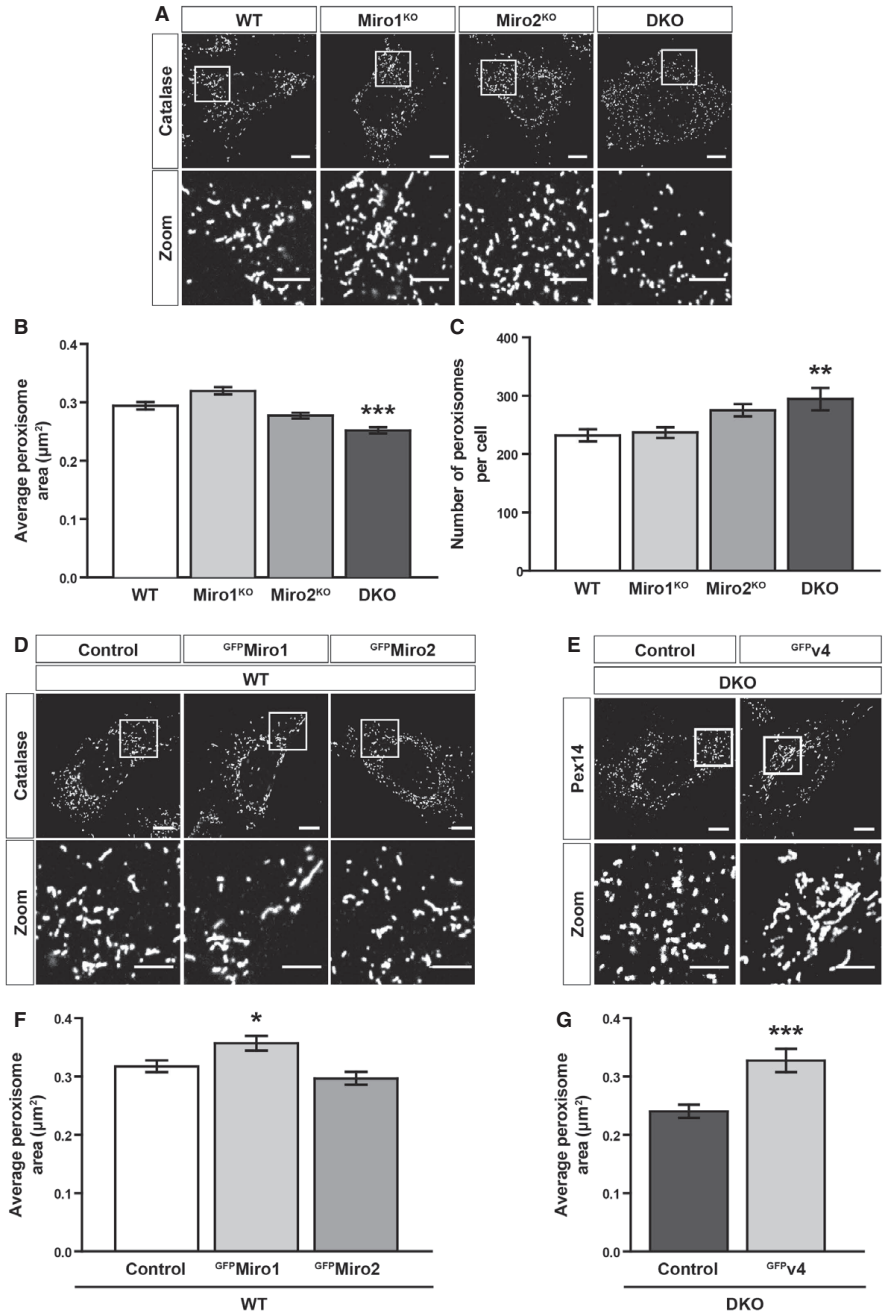
Miro regulates peroxisomal size, morphology and number Representative images of catalase staining (peroxisomes) in WT, Miro1^KO^, Miro2^KO^ and DKO MEFs.Bar graph comparing average peroxisomal area of WT Miro1^KO^, Miro2^KO^ and DKO MEFs (*n* = 60 cells per condition over three independent experiments).Comparison of total peroxisomal number between WT, Miro1^KO^, Miro2^KO^ and DKO MEFs. *n* = 60 cells per condition over three independent experiments.Representative images of control (GFP‐tagged 1‐70 of Tom70), ^GFP^Miro1 and ^GFP^Miro2 in WT MEFs stained with catalase (peroxisomes).Representative images of Pex14 (peroxisomes) in DKO MEFs expressing either GFP (control) or GFP‐tagged variant 4 of Miro1 (^GFP^v4).Bar graph comparing average peroxisomal area of control, ^GFP^Miro1 and ^GFP^Miro2 overexpressing WT MEFs (*n* = 60 cells per condition over three independent experiments).Comparison of average area of individual peroxisomes between DKO MEFs expressing either GFP (control) or GFP‐tagged variant 4 of Miro1 (^GFP^v4) (*n* = 30 cells per condition over three independent experiments).Data information: Statistical test used in (G) was a two‐tailed Student's *t*‐test. Calculation of statistical significance in (B), (C) and (F) was a one‐way ANOVA with Newman–Keuls *post hoc*. *, ** and *** denote *P* < 0.05, *P* < 0.01 and *P* < 0.001, respectively. Data are represented as mean ± SEM. Scale bar is 10 μm. Scale bar in zooms is 5 μm.

**Figure EV5:**
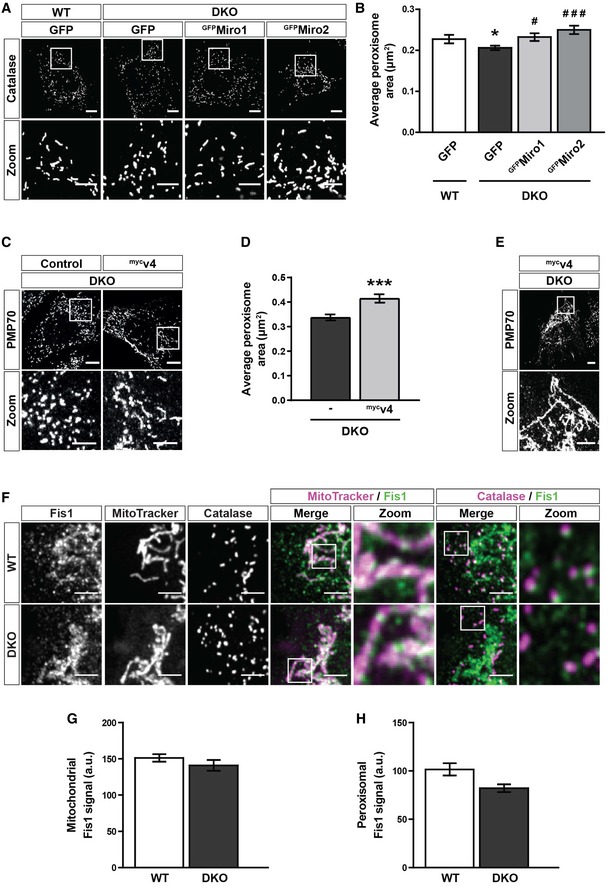
Re‐expression of Miro1 and Miro2 rescues peroxisomal size defect in DKO MEFs Representative images of catalase signal (peroxisomes) in WT MEFs and DKO MEFs either untransfected or expressing GFP‐tagged human Miro1 or Miro2 (^GFP^Miro1 and ^GFP^Miro2, respectively).Quantification of the average size of individual peroxisomes between WT, DKO, DKO‐expressing ^GFP^Miro1 and DKO‐expressing ^GFP^Miro2 (*n* = 36 cells per condition over three independent experiments).Representative images and zooms of peroxisomes (PMP70) of DKO MEFs and DKO MEFs transfected with ^myc^v4.Quantification of the average size of individual peroxisomes in DKO MEFs compared to DKO MEFs transfected with ^myc^v4 (*n* = 30 cells per condition over three independent experiments).Representative image and zoom of the long‐reticulated peroxisomal morphology observed in 20% of ^myc^v4‐expressing DKO cells.Zooms of endogenous Fis1, mitochondria (MitoTracker) and peroxisomes (PMP70) in WT and DKO MEFs.Integrated density of Fis1 signal on MitoTracker‐positive and PMP70‐negative structures in WT and DKO MEFs (*n* = 42 cells per condition over three independent experiments).Integrated density of Fis1 signal on PMP70‐positive and MitoTracker‐negative structures in WT and DKO MEFs (*n* = 42 cells per condition over three independent experiments).Data information: For (B) * denotes *P* < 0.05 in comparison with WT. ^#^ and ^###^ denote *P* < 0.05 and *P* < 0.001, respectively, in comparison with DKO MEFs by one‐way ANOVA with Newman–Keuls *post hoc* test. For (D), (G) and (H), statistical significance was tested with Student's *t*‐test. *** is *P* < 0.001. Scale bars are 10 μm, and 5 μm in zooms. Data are represented as mean ± SEM.

Miro has been shown to promote a long and reticulated mitochondrial morphology 54, 55, 56, 57. As the loss of Miro did not affect the basal distribution of peroxisomes, we next sought to investigate a potential role of Miro in peroxisomal size and morphology. Peroxisomes are known to adopt either a vesicular or tubular morphology with an average diameter ranging between 0.1 and 1 μm, depending on cell type and environmental cues 2. To explore whether Miro is required for the maintenance of peroxisomal morphology, WT, Miro1^KO^, Miro2^KO^ and DKO MEFs were fixed and stained with catalase. In doing so, WT MEFs were found to exhibit a mixture of rounded and tubular peroxisomes (Fig 5A). In contrast, peroxisomes in DKO MEFs appeared smaller with a more vesicular morphology. To determine whether Miro1/2 is required to regulate peroxisomal size, the average area of individual peroxisomes was quantified. Comparison of peroxisomal area between WT and DKO MEFs showed a significant decrease in peroxisomal size in the DKO MEFs (Fig 5A and B). Furthermore, alongside leading to smaller peroxisomes, the loss of Miro1/2 led to a significant increase in the number of peroxisomes per cell (Fig 5C). Exploring the role of either Miro1 or Miro2 in the single knockout MEFs showed that both Miro1 and Miro2 could compensate in their role in maintaining peroxisomal size, with no difference in peroxisomal area or number being observed in comparison with the WT MEFs (Fig 5A–C). Rescuing with either Miro1 or Miro2 in DKO MEFs confirmed this compensation, as both ^GFP^Miro1 and ^GFP^Miro2 could rescue peroxisomal area to that of WT cells (Fig EV5A and B). In contrast to the apparent redundancy observed in the single knockout MEFs, overexpression of Miro1 but not Miro2 in WT cells led to an increase in average peroxisome size (Fig 5D and F). It is therefore possible that Miro1 is the main driver of peroxisomal morphology but Miro2 can compensate. To test whether the ability of Miro to modulate peroxisomal size is a direct consequence of Miro localising to peroxisomes, the peroxisomally localised Miro1 splice variant (variant 4) was expressed in DKO MEFs. Importantly, both ^GFP^v4 and ^myc^v4 led to a substantial increase in peroxisomal area in comparison with the control transfected DKO MEFs (Figs 5E and G, and EV5C and D). Additionally, in variant 4‐expressing DKO MEFs we observed an extensive, reticulated peroxisomal network (reminiscent of the peroxisomal phenotype upon the loss of the fission machinery 8, 10, 11) in approximately 20% of cells (Fig EV5E). This phenotype was never observed in any experiment for untransfected DKO MEFs. Therefore, we conclude that Miro has a direct role in the maintenance of peroxisomal morphology and size, independent of its mitochondrial localisation.

**Figure 6:**
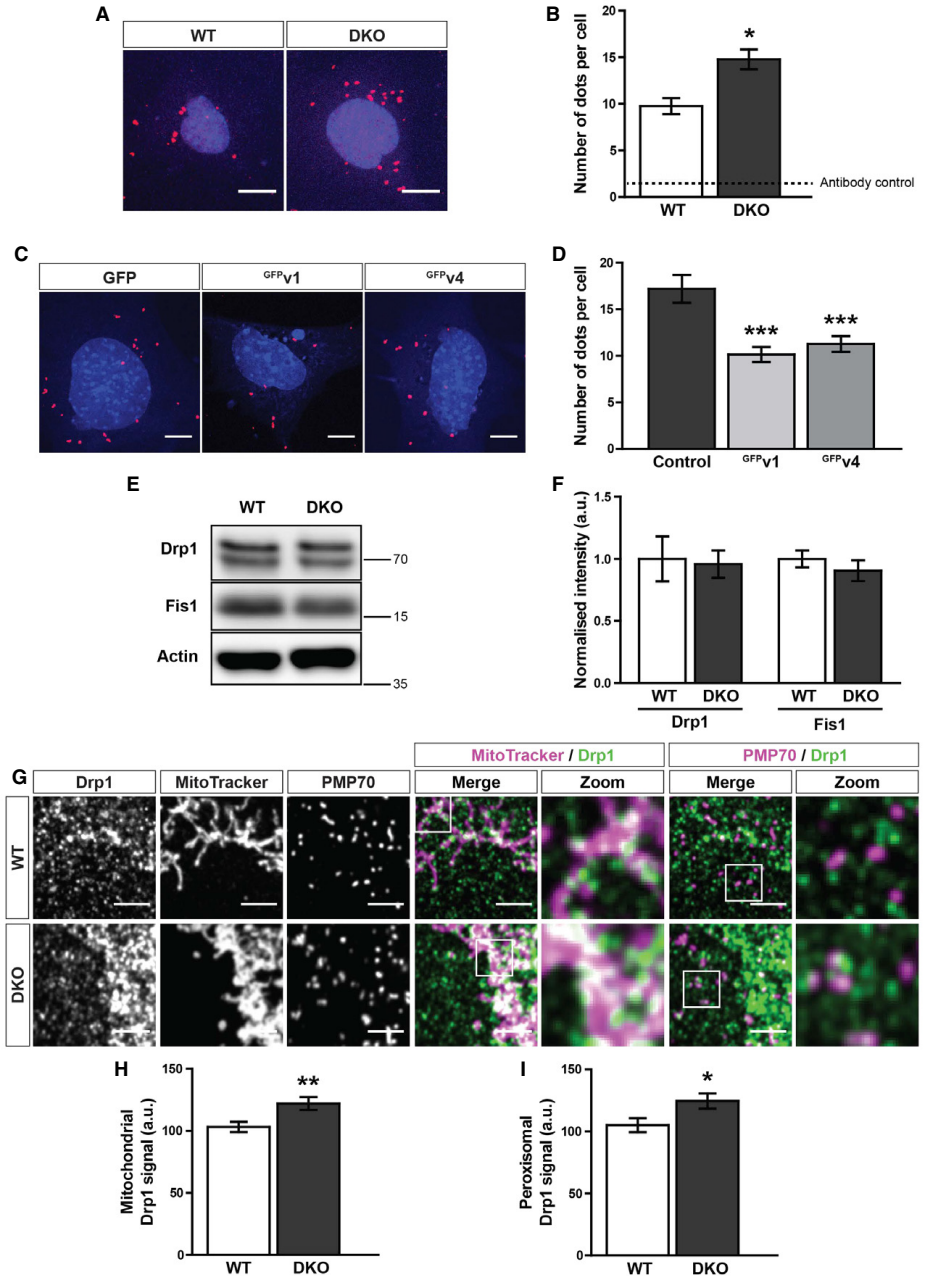
Miro negatively regulates the recruitment of Drp1 to peroxisomes and mitochondria Representative images of proximity ligation assay (PLA) of Fis1 and Drp1 in WT and DKO MEFs. Scale bar is 10 μm. Red dots indicate interaction of Fis1 and Drp1. Blue is DAPI.Quantification of fluorescent dots from PLA of Fis1 and Drp1 in WT and DKO MEFs. Dotted line indicates the sum of the Fis1 and Drp1 single antibody controls (*n* = 3 experiments).Representative images of a Fis1‐Drp1 PLA in DKO MEFs expressing either GFP, ^GFP^v1 (GFP‐tagged variant 1 of Miro1) or ^GFP^v4 (GFP‐tagged variant 4 of Miro1) (*n* = 30 cells per condition over three independent experiments). Scale bar is 10 μm. Red dots indicate interaction of Fis1 and Drp1. Blue is DAPI.Quantification of PLA fluorescent dots per cells between GFP, ^GFP^v1 or ^GFP^v4 transfected DKO MEFs (*n* = 30 cells over three independent experiments).Western blot of Drp1, Fis1 and actin from WT and DKO whole cell lysates.Quantification of normalised band intensity of Drp1 and Fis1 in WT and DKO MEFs (*n* = 3).Zooms of endogenous Drp1, mitochondria (MitoTracker) and peroxisomes (PMP70) in WT and DKO MEFs. Scale bar is 5 μm.Integrated density of Drp1 signal on MitoTracker‐positive and PMP70‐negative structures in WT and DKO MEFs.Integrated density of Drp1 signal on PMP70‐positive and MitoTracker‐negative structures in WT and DKO MEFs.Data information: (G–I) *n* = 42 cells per condition over three independent experiments. For (B), (F), (H) and (I), statistical significance was quantified by a two‐tailed Student's *t*‐test. In (D), a one‐way ANOVA with a Newman–Keuls *post hoc* test was used to calculate statistical significance. *, ** and *** denote *P* < 0.05, *P* < 0.01 and *P* < 0.001, respectively. Data are represented as mean ± SEM.

As peroxisomes were found to exhibit a smaller, more rounded morphology in the DKO MEFs—in addition to being more numerous—we hypothesised that Miro might be regulating peroxisomal fission. Importantly, mitochondria and peroxisomes have both been shown to use a Drp1‐dependent mechanism for fission, utilising the receptors Fis1 and Mff to recruit Drp1 from the cytoplasm 8, 9, 11, 58. We reasoned that Miro may negatively affect the recruitment of Drp1 from the cytoplasm and therefore the DKO MEFs could show an increase in interaction between Drp1 and Fis1. To explore this idea, we studied the Drp1‐Fis1 interaction *in situ*, using a proximity ligation assay (PLA) 59, 60. Briefly, if two proteins are closer than 40 nm (i.e. they associate), then fluorescence is amplified at that site, allowing a count of interactions per cell by the number of fluorescent dots. By studying the Drp1‐Fis1 interaction in WT and DKO MEFs by PLA, we found an approximately 50% increase in dot number per cell in the DKO MEFs in comparison with WT cells (Fig 6A and B). Crucially, expression of either the predominantly mitochondrial (variant 1) or peroxisomal (variant 4) splice variant of Miro1 in the DKO MEFs led to a significant reduction in dot number in comparison with control transfected cells (Fig 6C and D). Two possible explanations for the increase in Drp1‐Fis1 interaction upon the loss of Miro are (i) an increase in expression of either Drp1 or Fis1; and (ii) an increase in Drp1 recruitment to both the mitochondrial and peroxisomal membranes. Quantification of whole cell lysates by Western blotting showed no difference in Drp1 and Fis1 levels between WT and DKO MEFs (Fig 6E and F). Furthermore, quantification of the extent of Fis1 on either mitochondria or peroxisomes by immunofluorescence also revealed no significant difference between the two genotypes of MEFs (Fig EV5F–H). Importantly, however, there was a significant enrichment of endogenous Drp1 at both peroxisomes and mitochondria observed in the DKO MEFs (Fig 6G–I). Therefore, Miro modulates peroxisomal and mitochondrial morphology through negatively regulating the recruitment of Drp1 by receptors such as Fis1.

## Discussion

In summary, we have identified a novel role for Miro in the regulation of peroxisomal fission. Through an interaction with Pex19, Miro localises to peroxisomes by a mechanism dependent on its transmembrane domain and signalling through the first GTPase domain, exon 19 and exon 20. Our data suggest that Miro is not critical for steady‐state microtubule‐dependent trafficking and distribution of peroxisomes, but rather is required for the maintenance of peroxisomal size and morphology. Mechanistically, this occurs through negatively regulating the recruitment of Drp1 to the peroxisomal membrane. Interestingly, this function is not isolated to peroxisomal Miro but is shared with mitochondria, highlighting an overarching mechanism for control of these two metabolic organelles by Miro.

One question that arises from the dual localisation of Miro to mitochondria and peroxisomes is how are the relative pools of Miro on each organelle achieved? Recently, Okumoto *et al* 26 provided evidence that alternative splicing of exon 19 in human Miro1, at a site near the transmembrane domain, leads to its enhanced peroxisomal localisation. Interestingly, from their *in vitro* binding assays, they propose that Miro1 is targeted to peroxisomes by exon 19 sequences interacting with Pex19. Our data, however, show that Miro proteins lacking the exon 19 splice cassette—including Miro2 and the most common Miro1 variant (variant 1)—can also be found targeted to peroxisomes, in agreement with an analysis of C‐terminally anchored proteins 24. In agreement with this, we show the transmembrane domain of Miro, which is common to all Miro variants, is necessary for the interaction with Pex19 in cells, and by extension the localisation of Miro to peroxisomes. Indeed, Pex19 has been shown to bind the transmembrane domain of its targets 39, 40. Moreover, the subcellular localisation of C‐terminally anchored proteins is well documented as being dependent on the biochemical properties of the transmembrane domain and C‐terminal amino acids 24, 40, 61, 62.

Though Pex19 is known to be the chaperone required for the peroxisomal targeting of C‐terminally anchored proteins (such as Miro), the targeting of C‐terminally anchored proteins to the mitochondria is less well understood. Recent work in yeast has identified the cytosolic chaperones ssc1 and ssc2 as potential factors required for targeting of C‐terminally anchored proteins to the OMM 63. Interestingly, Cichocki *et al* 63 also show that the loss of Pex19 impinges upon mitochondrial targeting. Our work, in conjunction with Okumoto *et al* 26, shows that several amino acid sequences within Miro1 alter the relative mitochondrial–peroxisomal targeting, namely exon 19, exon 20, the transmembrane domain and the first GTPase domain. With this in mind, we propose the following model: the Miro transmembrane domain is required for Pex19 binding and peroxisomal localisation of Miro. Therefore, other features such as the first GTPase domain and sequences within exon 19/20 may act as important sites for regulatory factors and chaperones to bind and modulate the extent of mitochondrial and peroxisomal localisation. Consequently, the ability to control the extent of the mitochondrial and peroxisomal localisation of Miro may be an important regulatory axis; an axis that likely includes members of an ever‐growing list of proteins targeted to both organelles including USP30, Fis1, Mff, MUL1/MAPL, OMP25, BCL‐XL, BCL‐2, MAVS and GDAP1 8, 10, 21, 22, 23, 24, 25. The differential localisation of the variants of Miro uncovered here and in Okumoto *et al* 26 may therefore provide a useful set of tools for uncovering chaperones and regulatory proteins required for the targeting of proteins to the mitochondrial and peroxisomal membranes.

Miro plays an important role in establishing a properly distributed mitochondrial network in many cell types through long‐range microtubule‐dependent trafficking 30, 31, 49, 50. Indeed, we have shown that genetically knocking out all Miro1 and Miro2 in MEFs halts long‐range mitochondrial trafficking leading to a dramatic perinuclear collapse of the mitochondrial network 31. In line with the role of Miro in mitochondrial trafficking, it has been reported that peroxisomally localised Miro1 can modulate long‐range trafficking and the subsequent redistribution of peroxisomes 26, 34. Here, however, using a combination of micropattern‐based cell standardisation and quantitative organelle distribution analysis, we now show that compared to the marked redistribution of mitochondria in Miro1^KO^ and Miro1/2 DKO MEFs, steady‐state peroxisomal distribution remains unaffected. In addition, whilst we have previously shown that knocking out all Miro leads to a dramatic reduction in directed microtubule‐dependent transport of mitochondria, we found no change in the number and length of long‐range microtubule‐dependent peroxisomal trafficking events in the absence of Miro. This holds true for the acute and chronic loss of Miro1 and the complete loss of both Miro paralogues, ruling out any compensatory role by Miro2. Thus, unlike for mitochondria, we find that the primary role of Miro on peroxisomes does not appear to be to mediate their steady‐state distribution throughout the cell. In contrast, we find that the loss of both Miro1 and Miro2 in the DKO MEFs leads to a significant reduction in the shorter‐range displacements of peroxisomes, a type of trafficking that makes up ~90% of all peroxisomal movement 15, 17, 45. We show that the short‐range transport of peroxisomes is associated with the ER in both WT and DKO MEFs; however, we are currently uncertain as to the causality and mechanism of this movement. Given that knockout of Miro2 alone leads to reductions in short‐range peroxisomal displacements and that Miro2 is also localised to the ER 24, it could be that this reduction in peroxisomal transport is a downstream effect of Miro2 on ER dynamics.

As we tested trafficking in both knockout and knockdown of Miro in mouse and human cells, respectively, it is possible that the differences between our conclusions and those made in Okumoto *et al* 26 and Castro *et al* 34 arise from differences in analysis. Both papers use automatic tracking in conjunction with a velocity and track length cut‐off, in comparison with our blind scoring. There is a potential issue that arises from using velocity and track length cut‐off: the persistent directionality of microtubule‐dependent transport is not considered (whereas it is in blind scoring). This is particularly confounding when considering that it is assumed that short‐range peroxisomal trafficking is slow. It is the case that the early work characterising peroxisomal trafficking reported that short‐range peroxisomal displacements were slow oscillatory motions 15, 16. However, in comparison with those early studies, modern microscopy techniques allow for higher temporal resolution and though the net movement of these short‐range displacements is low, they can move at high velocities and with long track lengths. Indeed, Castro *et al* 34, 52 show a general shift in peroxisomal velocity, not just in what they define as long‐range transport. As a result, we believe that Miro primarily has an effect on short‐range trafficking rather than basal long‐range, microtubule‐dependent transport. It is important to note here that whilst our loss‐of‐function experiments show no change in basal long‐range peroxisomal transport, it is possible that there are conditions where Miro can alter peroxisomal distribution through microtubule‐directed transport. Both Okumoto *et al* 26 and Castro *et al* 34, 52 show changes in peroxisomal distribution upon overexpression of peroxisomally targeted Miro1. It would therefore be important to determine whether physiologically relevant circumstances exist where Miro can drive alterations in peroxisomal positioning by long‐range trafficking; however, in stark contrast to the crucial role of Miro for regulating mitochondrial distribution, we find that Miro is not required for maintaining steady‐state peroxisomal distribution.

Both overexpression and loss‐of‐function studies of Miro have shown that Miro promotes an elongated mitochondrial morphology 31, 55. Through overexpression, knockout and rescue studies, we find that Miro has a direct regulatory role in the control of peroxisomal size, number and morphology, supporting the idea that Miro has a shared morphological function at both mitochondria and peroxisomes 31, 55. Mechanistically, we find that the ability of Miro to modulate peroxisomal and mitochondrial size and morphology is through negatively regulating the recruitment of Drp1 by Fis1. Strikingly, in a subset of variant 4‐expressing cells, the peroxisomes formed a long, reticulated network reminiscent of the phenotype observed upon the loss of the fission machinery 8, 10, 11. Indeed, Drp1 activity has been shown to be sufficient for the scission of both mitochondria and peroxisomes, making the recruitment of Drp1 to membranes an essential step in fission 11. It should be noted that Mff, another Drp1‐receptor, is also localised to peroxisomes and therefore the effect of Miro in Mff‐dependent Drp1 recruitment should also be considered 8. Furthermore, building a model for Miro in modulating peroxisomal morphology should also consider that Castro *et al* 34, 52 proposed that Miro1 might promote peroxisomal elongation through coupling to microtubules. Whilst we find a role for Miro in peroxisomal fission, the multifaceted role of Miro in mitochondrial dynamics, function and turnover 27, 64, 65, 66, 67 highlights the need to probe further roles for Miro at peroxisomes. For example, Gem1 (the yeast orthologue of Miro) has been identified as a potential regulator of mitochondria–peroxisome contact sites 68. Therefore, it is possible that Miro has a similarly diverse role at peroxisomes.

One important consideration from this work is why would Miro have a regulatory role at both peroxisomes and mitochondria? In fact, more broadly, why do peroxisomes and mitochondria share a number of proteins critical to the morphology and turnover of both organelles (e.g. USP30, Mff, Fis1)? Mitochondria and peroxisomes overlap in several key functions and have been suggested to be evolutionarily related 69. For example, both are sites of fatty acid β‐oxidation and lipid synthesis and have a role in ROS metabolism. The case could therefore be that the dynamics of both organelles must be co‐modulated to control optimal cellular metabolism. As a result, the ratio of mitochondrial to peroxisomal localisation of proteins (including Miro) could act as a means to coordinate the function of both organelles in a dynamic cellular environment.

## Materials and Methods

### Antibodies and reagents

#### DNA constructs

^GFP^Miro1 and ^GFP^Miro2 were cloned from ^myc^Miro1 and ^myc^Miro2 (described previously 32) into pEGFP‐C1; ^GFP^Tom70(1‐70), amino acids 1‐70 of human Tom70, were cloned into pEGFP‐N1; Miro1 truncation constructs were cloned from ^GFP^Miro1: ^GFP^ΔGTP1 (184–618 only), ^GFP^GTP1 (1–177 fused to 562–618) and ^GFP^GTP2 (412–618 only); ^myc^Miro1ΔTM cloned from ^myc^Miro1 (deletion of 593–618), pxDsRed from Addgene (#54503), pxGFP from Addgene (#54501), ER‐DsRed from Addgene (#55836) 70; and ^myc^Pex19 mouse Pex19 (NM_023041) cloned into pRK5‐myc vector. GFP‐tagged mouse Miro1 splice variants (^GFP^v1, ^GFP^v2, ^GFP^v3 and ^GFP^v4) were cloned from NM_021536 (v1), NM_001163354 (v2) and NM_001163354 (v3) (OriGene: MR209606, MR224107 and MR224933, respectively) into pEGFP‐N1. ^GFP^v4 was made from ^GFP^v2 and ^GFP^v3 by Infusion cloning (TaKaRa). Myc‐tagged Miro1 mouse variants were cloned from their corresponding GFP‐tagged versions.

#### siRNA oligos

*Miro1*: 5′‐UAACCAAAUCGUCGAAGCACAGUCC‐3′ 26 and *Pex14* as a pool of four oligos: (i) 5′‐GAACUCAAGUCCGAAAUUA‐3′; (ii) 5′‐CCUCAUAUCUCAGCCAUAC‐3′; (iii) 5′‐CCAGACAGUGACUCAGUUA‐3′; and (iv) 5′‐AGGCAUUGCAUUUGGCUUU‐3′ 71. Oligos were transfected using Lipofectamine 3000 as per the manufacturer's instructions and left to express for 48 h.

#### Antibodies

For immunofluorescence (IF) and Western blotting (WB), primary antibodies were as follows: rabbit anti‐Tom20 (Santa Cruz sc‐11415, IF 1:500), mouse anti‐Catalase (Abcam ab110292, IF 1:500), rabbit anti‐Pex14 (Atlas HPA04386, IF 1:500), rabbit anti‐PMP70 (Abcam ab109448, IF 1:1,000), mouse anti‐Drp1 (BD Biosciences 611113, IF 1:500, WB 1:1,000), rabbit anti‐Fis1 (Abcam ab96764, IF and WB 1:1,000), mouse anti‐myc supernatant (purified in house from 9E10 hybridoma cell line, WB 1:100), rat anti‐GFP (Nacalai Tesque 04404‐84, IF 1:2,000), rabbit anti‐Pex19 (Abcam ab137072, WB 1:1,000), mouse anti‐β‐tubulin (Sigma T5293, IF 1:500) and rabbit anti‐GFP (Santa Cruz sc‐8334, WB 1:100). Fluorescent secondary antibodies (all from Thermo Fisher Scientific, 1:1,000) were as follows: donkey anti‐rat Alexa Fluor 488 (A21208), goat anti‐rabbit Alexa Fluor 555 (A21430), donkey anti‐mouse Alexa Fluor 647 (A31571). MitoTracker Orange CMTMRos was obtained from Thermo Fisher Scientific (M7510). For STED super‐resolution microscopy, goat anti‐rabbit Alexa Fluor 594 (Thermo Fisher Scientific A‐11012 at 1:200) and goat anti‐mouse STAR‐RED (Abberior 1:200) were used.

### Cell lines

WT, Miro1^KO^, Miro2^KO^ and DKO MEFs were characterised previously 31. Miro1‐floxed ERT‐Cre‐recombinase MEFs were generated from E8.5 mouse embryos as previously described 31. Knockout of Miro1 was achieved by treating with 1 μM of 4‐OH tamoxifen for 48 h. For peroxisomal distribution analysis, MEFs were seeded onto large Y‐shaped fibronectin‐micropatterned coverslips (CYTOO 10‐012‐00‐18) at a density of 15,000–20,000 cells/cm^2^. Cells were then left to attach for three hours and then fixed for 10 minutes with 4% paraformaldehyde (PFA). Immunocytochemistry was then carried out as described below.

### Co‐immunoprecipitation and Western blot analyses

Cells were lysed in buffer containing 50 mM Tris–HCl pH 7.5, 0.5% Triton X‐100, 150 mM NaCl, 1 mM EDTA, 1 mM PMSF and protease inhibitor cocktail for 45 min at 4°C with rotation. Lysates were then centrifuged at 21,000 *g* for 15 min and the supernatant collected for inputs and subsequent immunoprecipitation. GFP‐tagged proteins were pulled down with GFP‐trap agarose beads (ChromoTek, gta‐10) for 2 h. Beads were then washed three times with the lysis buffer. Samples were run on SDS–PAGE gel and transferred onto nitrocellulose membrane. Membranes were blocked with 4% (w/v) milk in PBS with 0.1% Tween 20 (PBST). Primary antibodies were incubated overnight at 4°C, washed three times with PBST and incubated with the secondary for 45 min at room temperature. Following three washes with PBST, the membrane was developed by exposure to ECL substrate (Millipore, WBLUR0500) and imaged on the ImageQuant LAS4000 mini (GE Healthcare).

### Fixed imaging

Cells were fixed with 4% PFA (diluted in PBS for confocal imaging or in 80 mM PIPES, 1 mM EGTA and 1 mM MgCl_2_ at pH 6.8 for STED microscopy) for 10 min and blocked for 30 min with 10% horse serum, 5 mg/ml bovine serum albumin and 0.2% Triton X‐100 in PBS. Samples were stained with primary and secondary antibodies for 1 h each and imaged on a Zeiss LSM700 confocal using a 63× oil objective (NA = 1.4). STED super‐resolution microscopy was carried out using Abberior Instruments STEDYCON microscope using a Zeiss 100× oil objective.

### Proximity ligation assay

Cells were fixed with 4% PFA for 10 min and then permeabilised with 0.1% Triton X‐100 in PBS for 15 min. A proximity ligation assay to assess protein–protein interaction was carried out with the Duolink *in situ* red mouse/rabbit kit (Merck) as per the manufacturer's instructions. Anti‐Drp1 (1:300, BD Biosciences) and Anti‐Fis1 (1:1,000 Abcam) antibodies were incubated for one hour at room temperature at the appropriate primary antibody step. For WT vs. DKO, five fields of view were confocal‐imaged with a Zeiss 40× oil objective (NA = 1.3) with approximately 15 cells per image for each experimental repeat. The number of red dots was then divided by the number of nuclei to obtain an average dot number per cell. For transfected cells, images of individual cells was taken with a Zeiss 63× oil objective (NA = 1.4). The number of dots was then counted.

### Live imaging

Live imaging of pxDsRed in WT and DKO MEFs, or WT and Miro1^KO^ hippocampal neurons, was carried out at 37°C whilst perfusing a solution of 10 mM glucose, 10 mM HEPES, 125 mM NaCl, 5 mM KCl, 2 mM CaCl and 1 mM MgCl at pH 7.4 by addition of NaOH, onto the coverslips. A 60× water objective on an Olympus BX60M microscope with an Andor iXon camera was used to acquire images. MicroManager software was utilised to control the microscope set‐up. PxDsRed was excited with an ET548/10× filter. To depolymerise microtubules, vinblastine was added at 1 μM for 1 hour prior to imaging. Cytochalasin‐D was used at 1 μg/ml for 30 min.

Dual‐organelle imaging and peroxisomal imaging following siRNA transfection were achieved using live spinning‐disc microscopy. To image the ER and peroxisomes, MEFs were transfected with ER‐DsRed and pxGFP, respectively, using Lipofectamine 2000 (Thermo Fisher 11668027). Transfected cells were then seeded into 3‐cm glass bottom dishes coated with fibronectin and imaged using an inverted Nikon TiE stand with a Yokogawa CSU‐X1 spinning‐disc scan head and Hamamatsu C9100‐13 EMCCD. Movies were obtained at 37°C with humidified 5% CO_2_ (two frames a second for 2 min for dual‐organelle and one frame a second for 5 min for siRNA in HeLa cells).

### Image analysis

Quantification of the extent of peroxisomal localisation was carried out in ImageJ. Using a mitochondrial mask (e.g. Tom20 or MitoTracker), all GFP signal that overlapped with the mitochondria was removed. Thresholded GFP signal that co‐localised with peroxisomes (catalase, Pex14 or PMP70) was then measured and divided by total GFP fluorescence. Peroxisomal morphology was measured by quantification of thresholded catalase signal in ImageJ. Both area and Feret's diameter were measured. Live trafficking of pxDsRed signal was quantified using TrackMate and MTrackJ 72. Only tracks that last lasted more than half the movie were used for analysis to prevent peroxisomes occurring more than once in the dataset. Long‐range trafficking events were quantified by blind scoring. Events were counted if they were longer than 2 μm in length and followed a persistent, directional trajectory.

ER displacement was quantified as previously published 31. Briefly, the relative change in ER pixels every 10 seconds was calculated by the thresholded signal from t0 seconds being subtracted from t10 s. This is then iterated for every 10 second interval (e.g. t20–t10 s). Graphed data are the average relative pixel change per cell.

### Statistical analysis

GraphPad prism was used to statistically analyse data. For comparisons between two conditions, a two‐tailed Student's *t*‐test was used. For multiple comparisons, either a one‐way ANOVA with a Newman–Keuls *post hoc* test or Kruskal–Wallis with a *post hoc* Dunn's correction was used as stated in the figure legends. Graphed data are presented as mean ± SEM.

## Author contributions

CC‐C, VST, GL‐D and JTK designed experiments. CC‐C, VST and GL‐D collected and analysed the results. GL‐D generated all MEFs and cloned the ^GFP^Tom70(1‐70) construct. NB cloned the Miro1 truncation constructs. JD wrote ImageJ macros for all automated analyses. CC‐C and JTK wrote the manuscript.

## Conflict of interest

The authors declare that they have no conflict of interest.

## Supporting information



Expanded View Figures PDF

Movie EV1

Movie EV2

Movie EV3

Movie EV4

Movie EV5

Movie EV6

Movie EV7

Movie EV8

Movie EV9

Movie EV10

Movie EV11

Movie EV12

Review Process File
