# Supplementary material for: Peroxisomal fission is modulated by the mitochondrial Rho‐GTPases, Miro1 and Miro2
Source: EMBO Rep. 2020 Jan 2;21(2):e49865. doi: 10.15252/embr.201949865 (PMC7001505; doi:10.15252/embr.201949865)
Supplement: Supplementary file 1 — Expanded View Figures PDF [file EMBR-21-e49865-s001.pdf]

Expanded View Figures

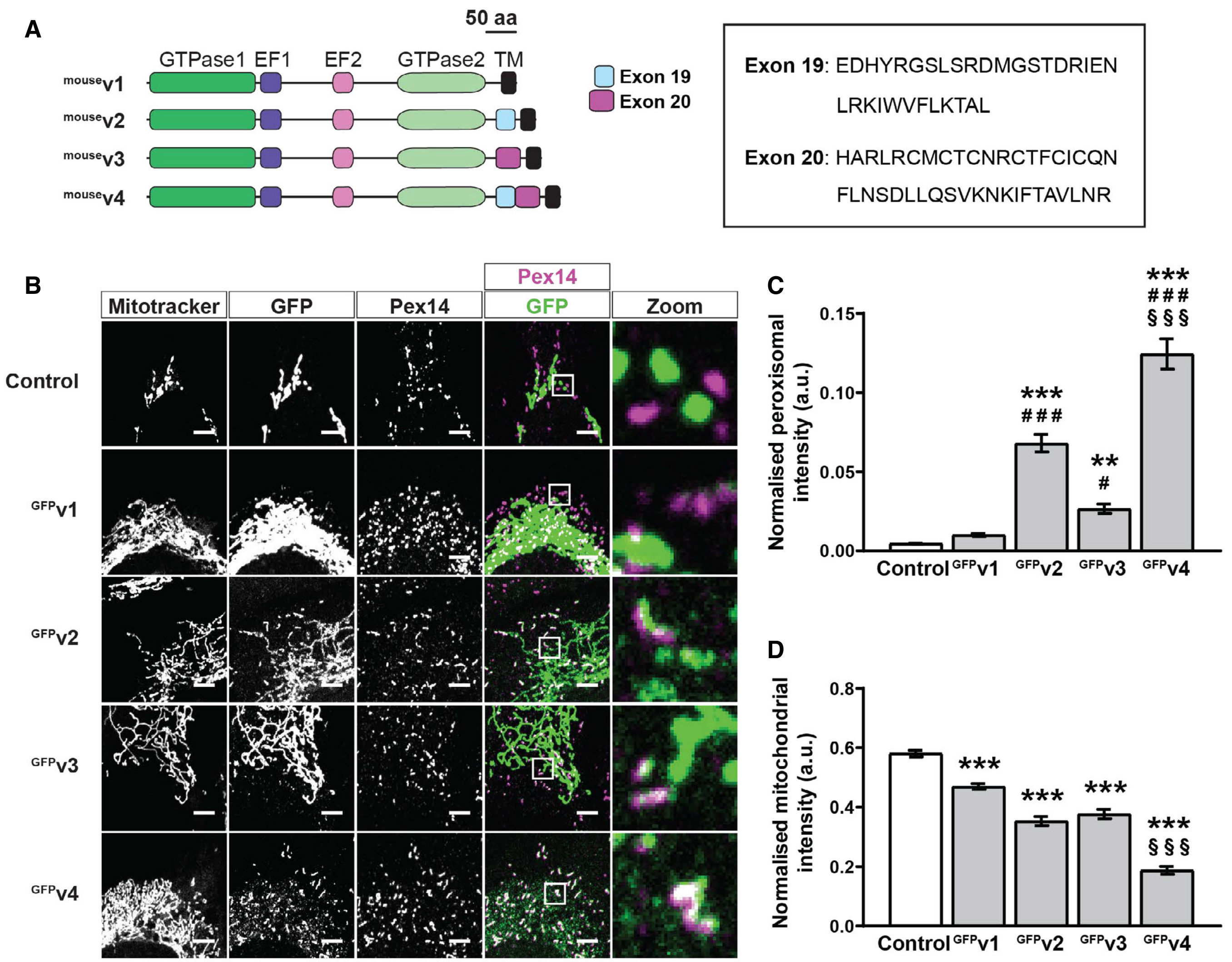

Figure EV1. Localisation of mouse Miro1 splice variants in DKOMEFs.

A Schematic of mouse Miro1 splice variants including amino acid sequences of exon 19 and exon 20. TM denotes the transmembrane domain.

B Representative images of DKO MEFs expressing control (GFP-tagged 1-70 of Tom70) or mouse Miro1 splice variants (^{GFP}v1, ^{GFP}v2, ^{GFP}v3 and ^{GFP}v4 corresponding to variants 1, 2, 3 and 4, respectively). MitoTracker stains mitochondria, and Pex14 stains peroxisomes. Scale bar is 5 µm.

C Comparison of Miro1 splice variant localisation to peroxisomes by thresholded GFP signal on Pex14-positive and MitoTracker-negative structures.

D Comparison of splice variant localisation to mitochondria by thresholded GFP signal on MitoTracker-positive and Pex14-negative structures.

Data information: For (C) and (D), n = 30 cells per condition over three independent experiments. ** denotes $P < 0.01$ in comparison with control. $^{\#}$ is $P < 0.05$ compared to ^{GFP}v1. ***, $^{\#\#\#}$ and §§§ denote $P < 0.001$ in comparison with control, ^{GFP}v1 and ^{GFP}v2, respectively, by one-way ANOVA with Newman–Keuls *post hoc* test. Data are represented as mean ± SEM.

© 2020 The Authors

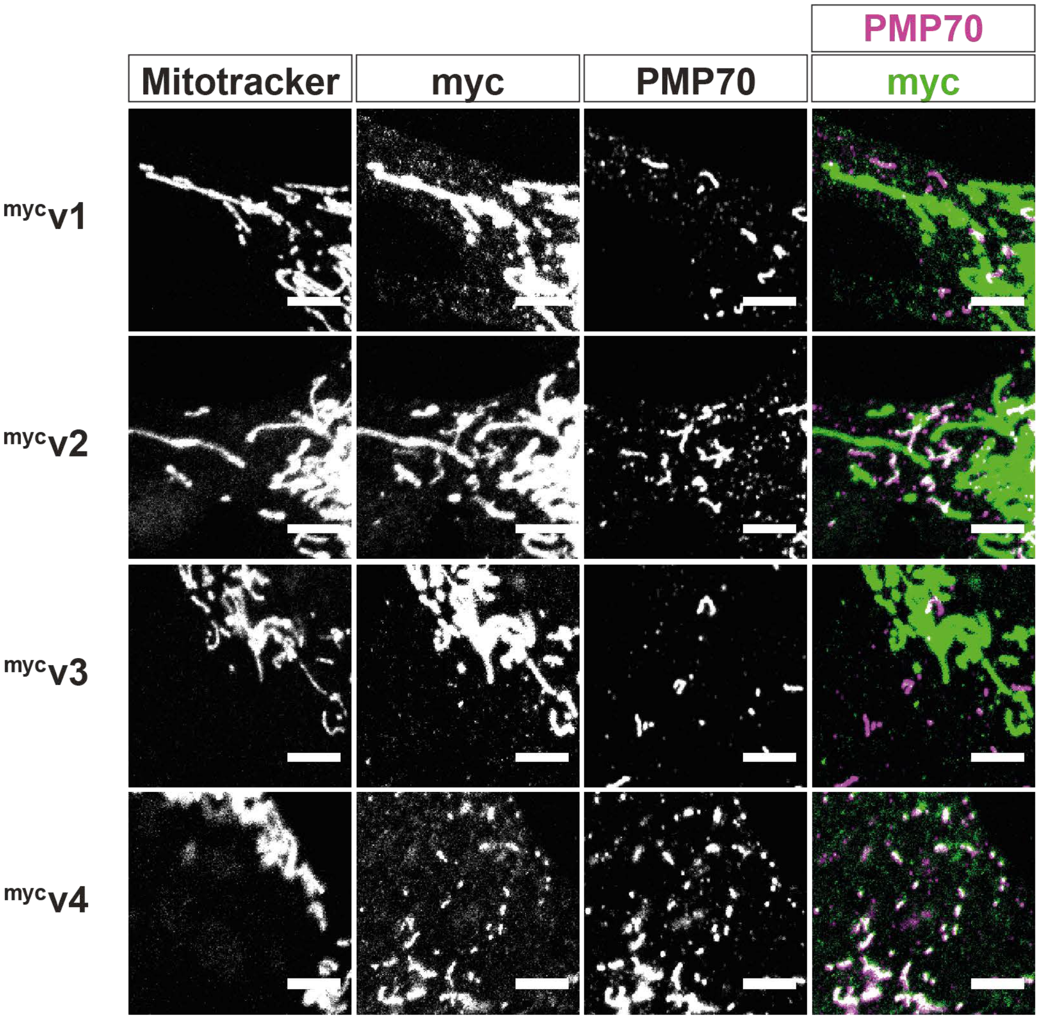

Figure EV2. Characterisation of myc-tagged mouse Miro1 splice variants.

Representative images of myc-tagged mouse Miro1 splice variants (^{myc}v1, ^{myc}v2, ^{myc}v3 and ^{myc}v4 corresponding to variants 1, 2, 3 and 4, respectively). MitoTracker stains mitochondria, and PMP70 stains peroxisomes. Scale bar is 5 μm.

© 2020 The Authors

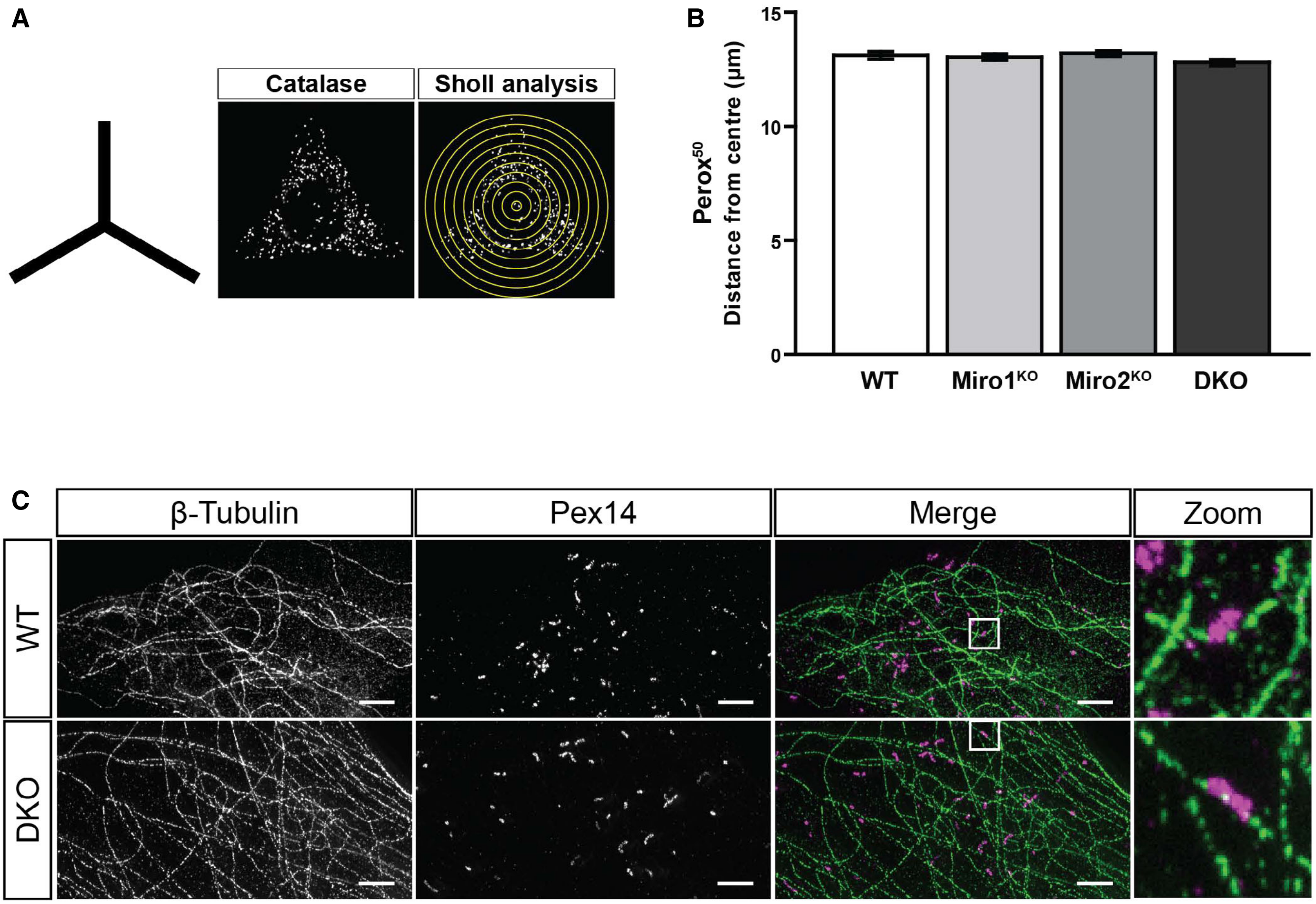

Figure EV3. Peroxisomes can still associate with microtubules following the loss of Miro.

A Schematic of the shape of the fibronectin patterns used for organelle distribution experiments and representative image of catalase signal of WT MEF on a micropattern along with a schematic of Sholl analysis.

B Quantification of the distance at which 50% of peroxisomal signal is situated in WT, Miro1^{KO}, Miro2^{KO} and DKO MEFs following Sholl analysis (n = 60 cells per condition over three independent experiments). No statistical significance was observed following a one-way ANOVA. All data are represented as mean ± SEM.

C Representative images of STED imaging of β-tubulin (microtubules in green) and Pex14 (peroxisomes in magenta) in WT and DKO MEFs. Scale bar is 2 µm.

© 2020 The Authors

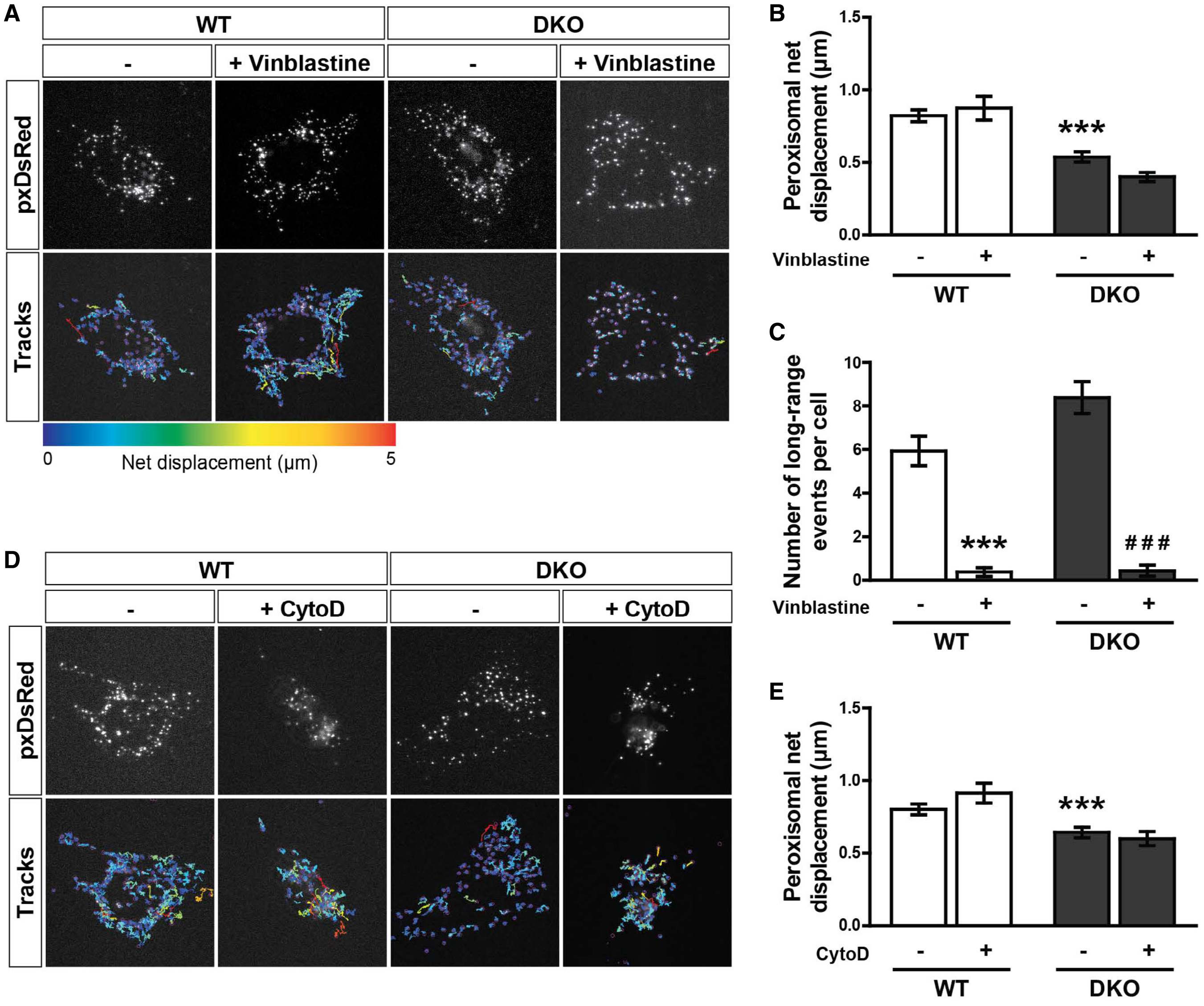

Figure EV4. The effect of perturbation to the microtubules and actin on peroxisomal motility.

A Snapshots and tracks from representative movies of peroxisome motility (pxDsRed) of untreated and vinblastine-treated (1 µM for 1 h) WT and DKO MEFs. Movies were acquired at 1.5 s per frame for 2 min.

B Median net displacement of peroxisomes over a 2-min period (n = 16 cells per condition over three independent experiments).

C Blind scoring of long-ranged peroxisomal trafficking events per cell over a 2-min movie. n = 16 cells per condition over three independent experiments.

D Snapshots and tracks from representative movies of peroxisome motility (pxDsRed) of untreated and cytochalasin-D (CytoD)-treated (1 µg/ml for 30 min) WT and DKO MEFs. Movies were acquired at 1.5 s per frame for 2 min.

E Median net displacement of peroxisomes from untreated and cytochalasin-D-treated WT and DKO MEFs (n = 18 cells per condition over three independent experiments).

Data information: Two-way ANOVA was used to test for significance; *** and $^{\#\#\#}$ denote $P < 0.001$ in comparison with WT untreated and DKO untreated MEFs, respectively. Data are represented as mean ± SEM.

© 2020 The Authors

Figure EV5. Re-expression of Miro1 and Miro2 rescues peroxisomal size defect in DKO MEFs.

- A Representative images of catalase signal (peroxisomes) in WT MEFs and DKO MEFs either untransfected or expressing GFP-tagged human Miro1 or Miro2 (^{GFP}Miro1 and ^{GFP}Miro2, respectively).
- B Quantification of the average size of individual peroxisomes between WT, DKO, DKO-expressing ^{GFP}Miro1 and DKO-expressing ^{GFP}Miro2 (*n* = 36 cells per condition over three independent experiments).
- C Representative images and zooms of peroxisomes (PMP70) of DKO MEFs and DKO MEFs transfected with ^{myc}v4.
- D Quantification of the average size of individual peroxisomes in DKO MEFs compared to DKO MEFs transfected with ^{myc}v4 (*n* = 30 cells per condition over three independent experiments).
- E Representative image and zoom of the long-reticulated peroxisomal morphology observed in 20% of ^{myc}v4-expressing DKO cells.
- F Zooms of endogenous Fis1, mitochondria (MitoTracker) and peroxisomes (PMP70) in WT and DKO MEFs.
- G Integrated density of Fis1 signal on MitoTracker-positive and PMP70-negative structures in WT and DKO MEFs (*n* = 42 cells per condition over three independent experiments).
- H Integrated density of Fis1 signal on PMP70-positive and MitoTracker-negative structures in WT and DKO MEFs (*n* = 42 cells per condition over three independent experiments).

Data information: For (B) * denotes $P < 0.05$ in comparison with WT. $^{\#}$ and $^{\#\#\#}$ denote $P < 0.05$ and $P < 0.001$, respectively, in comparison with DKO MEFs by one-way ANOVA with Newman–Keuls *post hoc* test. For (D), (G) and (H), statistical significance was tested with Student's *t*-test. *** is $P < 0.001$. Scale bars are 10 μm, and 5 μm in zooms. Data are represented as mean ± SEM.

© 2020 The Authors

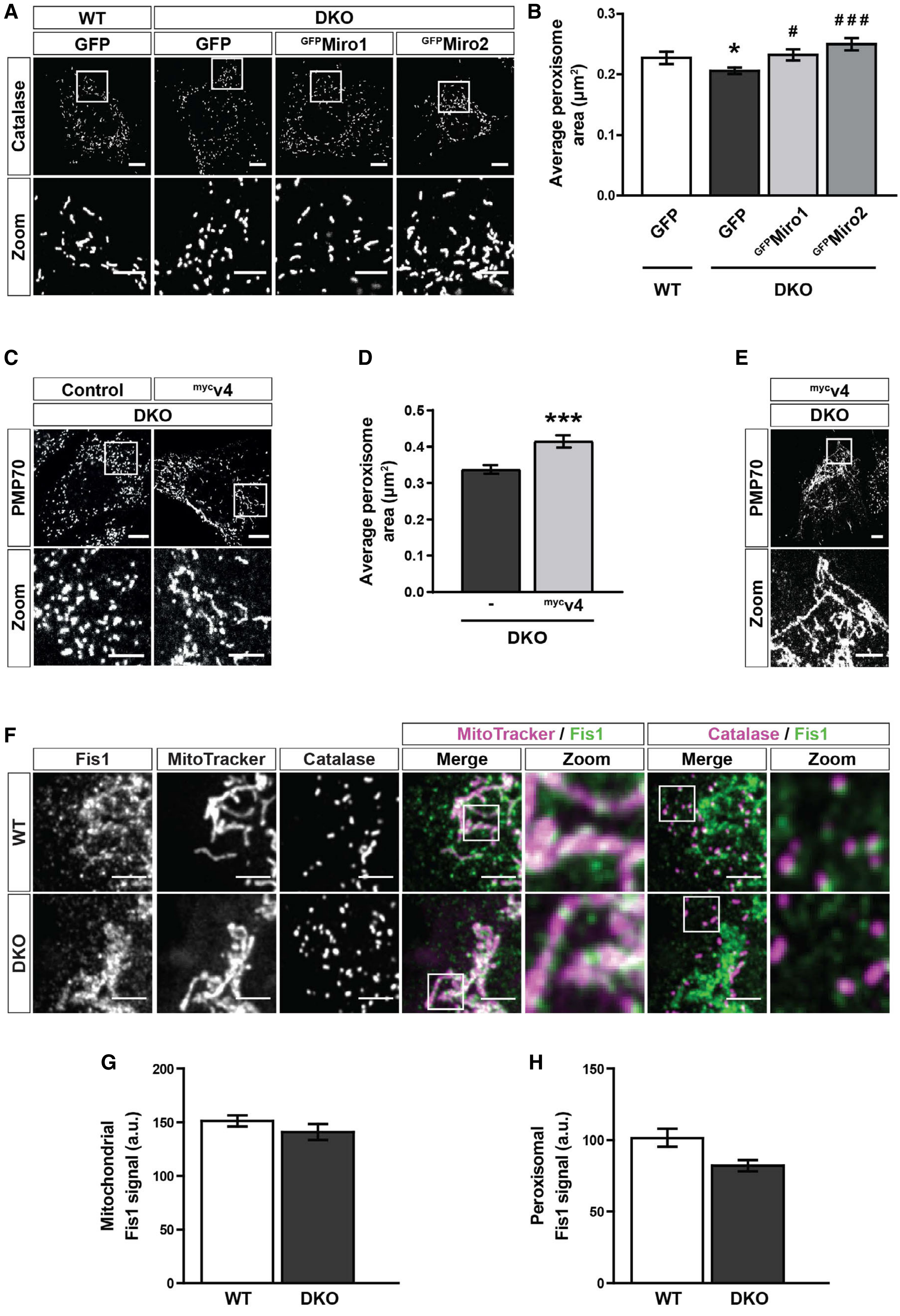

Figure EV5.

© 2020 The Authors
