# Supplementary material for: Peroxisomal fission is modulated by the mitochondrial Rho‐GTPases, Miro1 and Miro2
Source: EMBO Rep. 2020 Jan 2;21(2):e49865. doi: 10.15252/embr.201949865 (PMC7001505; doi:10.15252/embr.201949865)
Supplement: Supplementary file 9 — Movie EV8 [file EMBR-21-e49865-s009.zip › Movie_EV8.docx]

**Movie EV8: Representative movie of peroxisomal trafficking in vinblastine treated DKO MEFs**. Peroxisomal trafficking by imaging pxDsRed at 1.5 seconds per frame for two minutes. Cells were treated with 1 μM vinblastine for 1 hour before imaging.
