# Supplementary material for: Peroxisomal fission is modulated by the mitochondrial Rho‐GTPases, Miro1 and Miro2
Source: EMBO Rep. 2020 Jan 2;21(2):e49865. doi: 10.15252/embr.201949865 (PMC7001505; doi:10.15252/embr.201949865)
Supplement: Supplementary file 12 — Movie EV11 [file EMBR-21-e49865-s012.zip › Movie_EV11.docx]

**Movie EV11: Dual imaging of peroxisomes and ER in WT MEFs.** ER-DsRed (green) and pxGFP (magenta) imaged in WT MEFs at two frames a second for two minutes by spinning disk microscopy.
