# Supplementary material for: Peroxisomal fission is modulated by the mitochondrial Rho‐GTPases, Miro1 and Miro2
Source: EMBO Rep. 2020 Jan 2;21(2):e49865. doi: 10.15252/embr.201949865 (PMC7001505; doi:10.15252/embr.201949865)
Supplement: Supplementary file 13 — Movie EV12 [file EMBR-21-e49865-s013.zip › Movie_EV12.docx]

**Movie EV12: Dual imaging of peroxisomes and ER in DKO MEFs.** ER-DsRed (green) and pxGFP (magenta) imaged in DKO MEFs at two frames a second for two minutes by spinning disk microscopy.
