# Supplementary material for: Peroxisomal fission is modulated by the mitochondrial Rho‐GTPases, Miro1 and Miro2
Source: EMBO Rep. 2020 Jan 2;21(2):e49865. doi: 10.15252/embr.201949865 (PMC7001505; doi:10.15252/embr.201949865)
Supplement: Supplementary file 14 — Review Process File [file EMBR-21-e49865-s014.pdf]

EMBO *reports*

Peroxisomal fission is modulated by the mitochondrial Rho-GTPases, Miro1 and Miro2

Christian Covill-Cooke, Viktoriya S. Toncheva, James Drew, Nicol Birsa, Guillermo López-Doménech, and Josef T. Kittler

Review timeline:

	Submission date:	25 January 2018
	Editorial Decision:	13 March 2018
	Revision received:	31 March 2019
	Editorial Decision:	20 May 2019
	Revision received:	7 September 2019
	Editorial Decision:	16 October 2019
	Revision received:	8 November 2019
	Accepted:	13 November 2019

Editor: Martina Rembold

Transaction Report:

(Note: With the exception of the correction of typographical or spelling errors that could be a source of ambiguity, letters and reports are not edited. The original formatting of letters and referee reports may not be reflected in this compilation.)

1st Editorial Decision 13 March 2018

Thank you for the submission of your research manuscript to our journal. We have now received the full set of referee reports that is copied below. I apologize for the delay in handling your manuscript but we have also discussed it with an editorial advisor.

As you will see, both referees acknowledge that the findings are potentially interesting. However, referees 1 and 2 also point out several technical concerns and have a number of suggestions for how the study should be strengthened. Both referees indicate that further data are required to substantiate the conclusion that Miro1/2 affect the short-range, oscillatory movements of peroxisomes. Moreover, stronger evidence should be obtained that the loss of Miro1 and Miro2 does not affect long-range transport of microtubules in your setup.

From the referee comments it is clear that a major revision is necessary before the manuscript becomes suitable for publication in EMBO reports. However, given the overall positive evaluation and given the potential interest of your findings, we would like to give you the opportunity to address the concerns and would be willing to consider a revised manuscript with the understanding that the referee concerns (as detailed above and in their reports) must be fully addressed and their suggestions taken on board. Please address all referee concerns in a complete point-by-point response. Acceptance of the manuscript will depend on a positive outcome of a second round of review. It is EMBO reports policy to allow a single round of revision only and acceptance or rejection of the manuscript will therefore depend on the completeness of your responses included in the next, final version of the manuscript.

Revised manuscripts should be submitted within three months of a request for revision; they will otherwise be treated as new submissions. Please contact us if a 3-months time frame is not sufficient for the revisions so that we can discuss the revisions further.

You can submit the revision either as a Scientific Report or as a Research Article. For Scientific Reports, the revised manuscript can contain up to 5 main figures and 5 Expanded View figures. If the revision leads to a manuscript with more than 5 main figures it will be published as a Research Article. In this case the Results and Discussion section can stay as it is now. If a Scientific Report is submitted, these sections have to be combined. This will help to shorten the manuscript text by eliminating some redundancy that is inevitable when discussing the same experiments twice. In either case, all materials and methods should be included in the main manuscript file.

Supplementary/additional data: The Expanded View format, which will be displayed in the main HTML of the paper in a collapsible format, has replaced the Supplementary information. You can submit up to 5 images as Expanded View. Please follow the nomenclature Figure EV1, Figure EV2 etc. The figure legend for these should be included in the main manuscript document file in a section called Expanded View Figure Legends after the main Figure Legends section. Additional Supplementary material should be supplied as a single pdf labeled Appendix. The Appendix includes a table of content on the first page with page numbers, all figures and their legends. Please follow the nomenclature Appendix Figure Sx throughout the text and also label the figures according to this nomenclature. For more details please refer to our guide to authors.

Regarding data quantification, please ensure to specify the name of the statistical test used to generate error bars and P values, the number (n) of independent experiments underlying each data point (not replicate measures of one sample), and the test used to calculate p-values in each figure legend. Discussion of statistical methodology can be reported in the materials and methods section, but figure legends should contain a basic description of n, P and the test applied. Please also include scale bars in all microscopy images.

We now strongly encourage the publication of original source data with the aim of making primary data more accessible and transparent to the reader. The source data will be published in a separate source data file online along with the accepted manuscript and will be linked to the relevant figure. If you would like to use this opportunity, please submit the source data (for example scans of entire gels or blots, data points of graphs in an excel sheet, additional images, etc.) of your key experiments together with the revised manuscript. Please include size markers for scans of entire gels, label the scans with figure and panel number, and send one PDF file per figure.

When submitting your revised manuscript, we will require:

- a complete author checklist, which you can download from our author guidelines (http://embor.embopress.org/authorguide#revision). Please insert page numbers in the checklist to indicate where the requested information can be found.
- a letter detailing your responses to the referee comments in Word format (.doc)
- a Microsoft Word file (.doc) of the revised manuscript text
- editable TIFF or EPS-formatted figure files in high resolution
(In order to avoid delays later in the publication process please check our figure guidelines before preparing the figures for your manuscript:
http://www.embopress.org/sites/default/files/EMBOPress_Figure_Guidelines_061115.pdf)
- a separate PDF file of any Supplementary information (in its final format)
- all corresponding authors are required to provide an ORCID ID for their name. Please find instructions on how to link your ORCID ID to your account in our manuscript tracking system in our Author guidelines (http://embor.embopress.org/authorguide).

We would also welcome the submission of cover suggestions, or motifs to be used by our Graphics Illustrator in designing a cover.

As part of the EMBO publication's Transparent Editorial Process, EMBO reports publishes online a Review Process File to accompany accepted manuscripts. This File will be published in conjunction with your paper and will include the referee reports, your point-by-point response and all pertinent correspondence relating to the manuscript.

You are able to opt out of this by letting the editorial office know (emboreports@embo.org). If you do opt out, the Review Process File link will point to the following statement: "No Review Process File is available with this article, as the authors have chosen not to make the review process public in this case."

I look forward to seeing a revised version of your manuscript when it is ready. Please let me know if you have questions or comments regarding the revision.

****

REFEREE REPORTS

Referee #1:

Covill-Cooke et al. report the colocalization of Miro GTPases, known to be involved in mitochondrial transport on microtubules, on peroxisomes and show evidence of a function in short-distance oscillatory motion. First, using two cell types and GFP-tagged Miro1 or Miro2, they show that ~ 10-15% of Miro co-associates with peroxisomes, and it is likely through binding of the Miro1 TM with the Pex19 chaparone. The association with peroxisomes is increased in the absence of the first GTPase domain, and deleting this domain results in less Pex19 binding. Surprisingly, deletion of the two Miro paralogs does not change microtubule-based peroxisomal movement. Instead, deletion or overexpression modules short-distance oscillatory movement, and also peroxisomal shape.

It is intriguing that Miro shares function between mitochondria and peroxisomes and that this protein is involved in peroxisomal movement and shape. However, I found the data preliminary and less than compelling for the following reasons:

(1) As far as I can tell, the colocalization of Miro with peroxisomes are shown only upon overexpression of GFP-Miro. Peroxisomal localization would be more compelling if it can be shown at normal expression levels. Is Miro usually seen in peroxisomal proteomics studies? Can GFP be knocked into the chromosomal copy by CRISPR/Cas9 and peroxisomal localization still found?
(2) Related to this, it appears from the fluorescence data that only a subset of peroxisomes contains visible Miro. Do the peroxisomes with Miro have more oscillatory motion than those without?
(3) The effects of Miro knockout on mean velocity (Fig. 3 panel D) and peroxisomal area (Fig. 4 panel D) are fairly subtle. Again, is there is difference between peroxisomes with visible Miro and without?
(4) Most importantly, there is insufficient mechanistic information here. Is Miro tethering peroxisomes to the ER? Are we looking at active peroxisomal motion, or motion of the tethered host (ER?) Are the GTPases necessary for the motion?

There are a few minor points:

(1) In Fig. 1 panel C, virtually all of Miro1 is tethered to Pex19. Because it is not released from the chaperone, does Miro1 remain in the cytosol if Pex19 is overexpressed?
(2) A scale bar of amino acid # would be useful in Fig. 2 panel A. MW markers would be useful in Fig. 2 panel D.
(3) The contrast in the GFP channel in Fig. 2 panel B is not sufficient to assess the percent of peroxisomes that are tagged. Perhaps the single channel images can be black-and-white? I really don't have a good sense of the % of peroxisomes labeled with Miro.

Overall, the manuscript is interesting but lacks sufficient mechanistic data. It cites the recent JCB Okumoto paper showing peroxisomal Miro, but is contradictory regarding movement on microtubules. As it concludes a different mechanism for affecting movement (and even if the Okumoto paper did not exist), more data is necessary to underpin the authors' hypothesis.

Referee #2:

In their short report Covill-Cooke et al. provide evidence that the Miro family of proteins plays a

role in peroxisome mobility and morphology. Using MEF cells, they show that overexpressed Miro1 and Miro2 are localized to peroxisomes, and that this targeting requires their tail-anchored transmembrane domain and Pex19. They also suggest at least for Miro1, the first GTPase may negatively regulate Miro1 targeting to peroxisomes. Using quantitative analysis of high-resolution time-lapse imaging, they show that the long-range microtubule-dependent movement of peroxisomes is not affected by the loss of both Miro1 and Miro2, but rather their work suggests that the Miro protein may play a role in the shorter-range transport of peroxisomes. They also showed that double knockout Miro1/Miro2 cells has smaller peroxisomes, while overexpression of Miro1 in WT MEF cells results in larger peroxisomes.

This manuscript comes on the heels of two recent papers that also explore the role of Miro1 in peroxisome mobility, size and division. However, the manuscript by Covil-Cooke et al. present a couple novel findings including evidence that the first GTPase domain regulates Miro1 targeting, and that Miro2 localizes to peroxisomes. Furthermore, contrary to the finding by Okumoto et al JCB (2018) who has shown that Miro1 variants are required for the long-distance movement of peroxisomes, Covill-Cooke et al suggest that Miro proteins are required for short microtubule-independent movements. These findings are interesting and add to the field discussion regarding the regulation of peroxisome mobility and morphology. However, there are several concerns with the experiments and interpretation of the data.

1) Based on the observation of reduced GFP-GTP1 constructs on peroxisomes by confocal microscopy and decreased binding to Pex19, the authors suggest that the first GTPase domain may negatively regulate targeting of Miro1 to peroxisomes via Pex19. This is an over interpretation of the data since there are other more plausible interpretations. The lack of binding may rest on the truncated Miro1 construct itself and not on the GTP1 domain. Miro1 is a tail-anchored targeting protein where its membrane targeting signal is located at the TM and the sequence flanking it. Recently, Okumoto et. al (2017) JCB 217:619-33 have shown that the N-terminal region of the TM plays a role in targeting a Miro1 variant to peroxisomes, suggesting the importance of this region. From the method section, it is not clear how the GFP1 protein was constructed. However, based on the illustration in Figure 2A it appears that the GTP1 construct conside of the first 184 residues of Miro1 at the N-terminus of the TM domain. If this is indeed the case, the construct is likely missing the N-terminus flanking sequence that may be required to bind to Pex19. To test whether the GTP1 domain does regulate Pex19 binding, the authors should increase the length of their TM domain at least from the residue 560 to 618.

2) Total fluorescence intensity on peroxisomes is not a good indicator of quantitative measure of protein on peroxisomes (Figure 2C). Instead, the authors should compare subcellular fractionation to demonstrate a difference in the localization of the various constructs to peroxisomes.

3) The authors show that the double knock out of Miro1 and Miro2 results in the loss of short-distance movement, but has no effect in the microtubule long distance movement. This result conflicts from the recent work by Okumoto et al. One obvious difference between to two is the use of a double KO MEF versus the RNAi depletion of Miro1. Given that the Okumoto et al manuscript is published, the onus lays on this manuscript to explore the difference. Each system (KO vs RNAi) has their advantages and disadvantages. The authors should test whether they observe any differences in peroxisome mobility using the RNAi to deplete the cells of Miro1 and Miro2.

3) One novel aspect of this manuscript is the demonstration that Miro2 also targets to peroxisomes. However, the authors do very little to explore the role of Miro2. The authors should explore the role of Miro2 in respect to peroxisome mobility and size by testing whether expressing it in the double KO MEFs can rescue either the short distance movement or rescue its morphology.

4) Could it be possible that the smaller peroxisome size in the double KO cells be due to increased peroxisome division? In Figure 4C, there appears to be more peroxisomes in the DKO cells. The authors should quantify the number of peroxisomes in these cells.

5) Figure 1B and 2C: instead of measuring the area of GFP on peroxisomes, the authors need to measure the GFP on peroxisome compared to total peroxisome area. This is because the total amount or area of peroxisomes can differ between cells, and conditions.

1st Revision - authors' response 31 March 2019

Referee #1:

Covill-Cooke et al. report the colocalization of Miro GTPases, known to be involved in mitochondrial transport on microtubules, on peroxisomes and show evidence of a function in short-distance oscillatory motion. First, using two cell types and GFP-tagged Miro1 or Miro2, they show that ~ 10-15% of Miro co-associates with peroxisomes, and it is likely through binding of the Miro1 TM with the Pex19 chaparone. The association with peroxisomes is increased in the absence of the first GTPase domain, and deleting this domain results in less Pex19 binding. Surprisingly, deletion of the two Miro paralogs does not change microtubule-based peroxisomal movement. Instead, deletion or overexpression modules short-distance oscillatory movement, and also peroxisomal shape.
It is intriguing that Miro shares function between mitochondria and peroxisomes and that this protein is involved in peroxisomal movement and shape. However, I found the data preliminary and less than compelling for the following reasons:

(1) As far as I can tell, the colocalization of Miro with peroxisomes are shown only upon overexpression of GFP-Miro. Peroxisomal localization would be more compelling if it can be shown at normal expression levels. Is Miro usually seen in peroxisomal proteomics studies? Can GFP be knocked into the chromosomal copy by CRISPR/Cas9 and peroxisomal localization still found?
We agree that the presence of endogenous Miro on peroxisomes will strengthen our current model. In the literature, this has now been shown by at least four independent groups using a fractionation approach. Two of these used western blotting to show Miro1 in highly pure peroxisomal fractionation in both HeLa cells and liver (Costello *et al.*, 2017 and Okumoto *et al.*, 2018) and two used unbiased proteomic approaches on peroxisomal fractions (Wiese *et al.*, 2007 and Jadot *et al.*, 2017). Given the different approaches, across different species, we feel there is sufficient evidence to conclude Miro is resident at peroxisomes under basal conditions at endogenous levels. Our work therefore focuses on how this localisation can be regulated through amino acid sequences within Miro.

(2) Related to this, it appears from the fluorescence data that only a subset of peroxisomes contains visible Miro. Do the peroxisomes with Miro have more oscillatory motion than those without?
We propose that Miro1 and Miro2 are on all peroxisomes and not just localised to a subset. We have taken the reviewer's suggestion below (minor point 1) of displaying the single channel images in black and white and believe that this much more appropriately highlights our conclusion. Furthermore, we have looked at the integrated intensity of Miro1 signal on individual peroxisomes. These data show that the extent of Miro localisation to each peroxisome is predominantly influenced by the size of peroxisomes, as opposed to subpopulations of peroxisomes existing without Miro (Rebuttal figure 1). We have also cloned all four mouse Miro1 splice variants identified in Okumoto *et al.* (2018) and studied their localisation. As they exhibit a more prominent localisation to peroxisomes it is easier to see that all peroxisomes exhibit Miro localisation (Figure EV1). As a result, we propose Miro localises to all peroxisomes.

© European Molecular Biology Organization

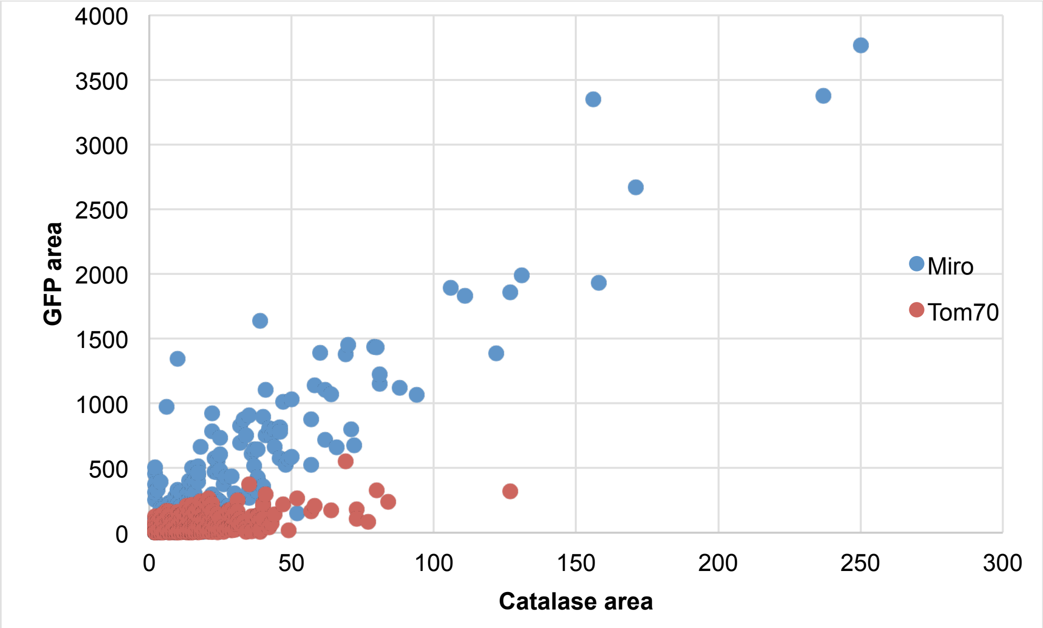

Rebuttal figure 1: Correlation of Miro1 signal to catalase (peroxisomal) signal.

(3) The effects of Miro knockout on mean velocity (Fig. 3 panel D) and peroxisomal area (Fig. 4 panel D) are fairly subtle. Again, is there is difference between peroxisomes with visible Miro and without?

Similarly to point 2) we believe Miro is on all peroxisomes. Therefore, it is not possible to compare the difference in behaviour between peroxisomes with and without Miro.

(4) Most importantly, there is insufficient mechanistic information here. Is Miro tethering peroxisomes to the ER? Are we looking at active peroxisomal motion, or motion of the tethered host (ER?) Are the GTPases necessary for the motion?

We thank the reviewer for their comment and agree that more mechanistic information would improve the manuscript. We have attempted to address this in a range of ways:

Firstly, the contribution of actin to short-range peroxisomal motility was explored by disruption of F-actin using cytochalasin D in both the WT and DKO MEFs. By comparing WT MEFs either untreated or treated with cytochalasin D no difference in short-range peroxisomal transport was observed (Figure EV2D-E). Importantly, the reduction in trafficking observed in the DKO MEFs was also unaffected, therefore ruling out the difference in trafficking between the WT and DKO MEFs being either active transport or anchoring on the actin cytoskeleton.

We next studied the role of the single knockout of either Miro1 or Miro2 on short-range peroxisomal transport. In doing so we found no difference between Miro1^{KO} and WT MEFs in this trafficking behaviour (Figure 3A-B). In contrast, however, we found a decrease in short-range peroxisomal displacements in Miro2^{KO} cells. As Miro2 has recently been found on the ER (Costello *et al.,* 2017), we probed the relationship of peroxisomal trafficking with the ER. To do so, dual-colour imaging of peroxisomes and ER was carried out by live-cell spinning disk microscopy. Strikingly, short-range (but not long-range) peroxisomal transport was significantly associated with the movement of the ER in the WT MEFs (Figure 3C; Supplementary movies 11). In fact, peroxisomes could be observed following the path of ER tubules. Importantly, peroxisomes in the DKO MEFs were also associated with the ER; with ER motility being significantly reduced (Figure 3D). We propose that the reduction in short-ranged peroxisomal transport in the DKO MEFs is likely caused by a reduction in ER dynamics.

Given the data from the dual-colour imaging experiments, we tested whether the reduction of peroxisomal trafficking is caused by a lack of Miro on peroxisomes, specifically. To do so, the peroxisomally localised splice variant of Miro1, variant 4, was expressed in DKO MEFs. Variant 4 of Miro1 did not rescue shorter-range peroxisomal displacements (Figure 3F), meaning that the localisation of Miro1 to peroxisomes alone is not sufficient for short-range peroxisomal trafficking. We therefore conclude that loss of all Miro causes defects in ER

© European Molecular Biology Organization

dynamics and that peroxisomal trafficking is associated with this movement. Localisation of Miro to peroxisomes is, however, a direct regulator of peroxisomal size, morphology and number (Figure 4 & 5), please see final point.

There are a few minor points:

(1) In Fig. 1 panel C, virtually all of Miro1 is tethered to Pex19. Because it is not released from the chaperone, does Miro1 remain in the cytosol if Pex19 is overexpressed?
Though the co-immunoprecipitated ^{myc}Miro1 signal in the blot appears at the same intensity as the input, the amount of input loaded is 1% of the total protein used for the IP. Therefore, most of the ^{myc}Miro1 will not be bound to Pex19. The figure has been updated to indicate this (Figure 1C). To ensure that the localisation of Miro is not drastically affected by the overexpression of Pex19, we expressed ^{GFP}Miro1 and ^{myc}Pex19 together. ^{GFP}Miro1 still exhibited a stereotypical Miro1 staining: predominantly mitochondrial with a small pool on peroxisomes (Rebuttal figure 2).

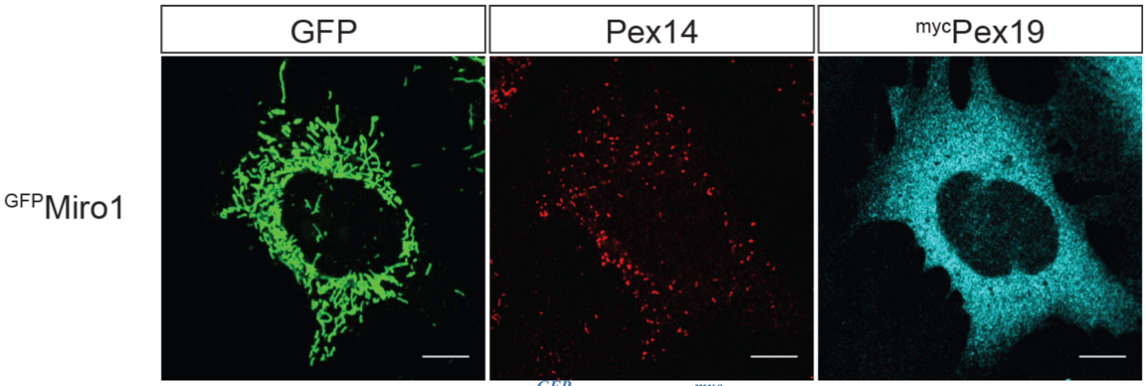

Rebuttal figure 2: Co-expression of ^{GFP}Miro1 and ^{myc}Pex19 in Cos7 cells.

(2) A scale bar of amino acid # would be useful in Fig. 2 panel A. MW markers would be useful in Fig. 2 panel D.
We thank the reviewer for their comment and agree a scale bar would be useful. The schematic for the Miro truncation constructs is now to scale and includes a scale bar (Figure 1D).

(3) The contrast in the GFP channel in Fig. 2 panel B is not sufficient to assess the percent of peroxisomes that are tagged. Perhaps the single channel images can be black-and-white? I really don't have a good sense of the % of peroxisomes labeled with Miro.
We thank the reviewer for this comment and agree that the images in the original manuscript did not properly represent our conclusions as well as they could. We have therefore changed the images to black and white (Figure 1A & E). Please also see the rebuttal to point 2).

Overall, the manuscript is interesting but lacks sufficient mechanistic data. It cites the recent JCB Okumoto paper showing peroxisomal Miro, but is contradictory regarding movement on microtubules. As it concludes a different mechanism for affecting movement (and even if the Okumoto paper did not exist), more data is necessary to underpin the authors' hypothesis.
We agree that the original manuscript would be improved by more mechanistic insight into the role of Miro in both peroxisomal trafficking and morphology. Furthermore, we have aimed to support our conclusion that Miro is not required for long-range, microtubule-dependent peroxisomal transport. We have addressed these points by the following:
1) To strengthen our case that Miro is not required for long-range, microtubule-dependent peroxisomal transport we have also quantified peroxisomal trafficking and distribution in Miro1 single knockout and Miro2 single knockout MEFs (characterised in López-Doménech *et al.*, 2018). We find no difference in long-range trafficking or peroxisomal distribution between any genotype (Figure 2 & EV2). In addition, since Okumoto *et al.* (2018) looked at the effect of acute loss of Miro1 on microtubule-dependent peroxisomal transport (using RNAi against Miro1) we have also studied the effect of acute loss of Miro1 in our system. This was achieved by tamoxifen treatment of Miro1 floxed MEFs expressing a tamoxifen-induced Cre-

recombinase. We show Miro1 is undetectable after three days following tamoxifen treatment (Figure EV2A). Upon quantification of long-range peroxisomal trafficking events, we found no difference between tamoxifen treated and control MEFs, meaning that the acute loss of Miro1 has no effect on peroxisomal trafficking (Figure EV2B). It is therefore unlikely that there are compensatory mechanisms masking the effect in the case of the chronic loss of Miro.
2) Through a series of live imaging assays we found that the reduction of short-range peroxisomal motility is elicited through interacting with the ER, see point 4).
3) The most important mechanistic development, however, is in our understanding of the role of Miro in regulating peroxisomal size and morphology. We have quantified peroxisomal size in Miro1 and Miro2 single knockout cells, showing that either Miro paralogue can compensate for the loss of the other. This is supported by both the expression of either ^{GFP}Miro1 or ^{GFP}Miro2 in DKO MEFs rescuing peroxisomal size. Importantly, we found that peroxisomally-targeted Miro1 (variant 4) can rescue the peroxisomal size defect in DKO MEFs (Figure 4E & G), leading to larger and more tubular peroxisomes. As a result, the presence of Miro at peroxisomes is required to modulate peroxisomal size and morphology.
Upon quantifying peroxisomal number we found that loss of Miro leads to a significant increase in peroxisomal number in the Miro DKO MEFs (Figure 4C). Given this, and the fact that peroxisomes cannot undergo fusion (Bonekamp *et al.*, 2012), we propose that Miro may regulate peroxisomal size and number through peroxisomal fission. Peroxisomal fission is elicited by the recruitment of Drp1 from the cytoplasm by Fis1, a process that is shared with the mitochondria (Koch *et al.*, 2003; Koch *et al.*, 2005; Kamerkar *et al.*, 2018). We hypothesise that Miro may negatively regulate the recruitment of Drp1 from the cytoplasm. To study this we developed a novel assay to test if there is an increase in the interaction with Drp1 with its receptor Fis1. This was achieved by a proximity ligation assay whereby interactions between two proteins of interest can be visualised *in situ*. By probing Drp1 and Fis1 we found a significant increase in Drp1-Fis1 interaction in DKO MEFs (Figure 5A-B). As no increase in either Drp1 or Fis1 levels was observed (Figure 5E-F) the prediction was that this is caused by the recruitment of more Drp1 from the cytoplasm to both organelles. Indeed, quantifying the extent of Drp1 at both peroxisomal and mitochondrial membranes showed that more Drp1 was localised to both organelles (Figure 5G-I). Expression of the peroxisomally-localised Miro1 splice variant (variant 4) reduced the interaction of Fis1 and Drp1 in the DKO MEFs, further highlighting the peroxisome specificity of the role of Miro in peroxisomal fission (Figure 5C-D). We therefore conclude that Miro modulates peroxisomal and mitochondrial size and morphology by negatively regulating the recruitment of Drp1 from the cytoplasm to the organelle membrane.

Referee #2:

In their short report Covill-Cooke et al. provide evidence that the Miro family of proteins plays a role in peroxisome mobility and morphology. Using MEF cells, they show that overexpressed Miro1 and Miro2 are localized to peroxisomes, and that this targeting requires their tail-anchored transmembrane domain and Pex19. They also suggest at least for Miro1, the first GTPase may negatively regulate Miro1 targeting to peroxisomes. Using quantitative analysis of high-resolution time-lapse imaging, they show that the long-range microtubule-dependent movement of peroxisomes is not affected by the loss of both Miro1 and Miro2, but rather their work suggests that the Miro protein may play a role in the shorter-range transport of peroxisomes. They also showed that double knockout Miro1/Miro2 cells has smaller peroxisomes, while overexpression of Miro1 in WT MEF cells results in larger peroxisomes.
This manuscript comes on the heels of two recent papers that also explore the role of Miro1 in peroxisome mobility, size and division. However, the manuscript by Covil-Cooke et al. present a couple novel findings including evidence that the first GTPase domain regulates Miro1 targeting, and that Miro2 localizes to peroxisomes. Furthermore, contrary to the finding by Okumoto et al JCB (2018) who has shown that Miro1 variants are required for the long-distance movement of peroxisomes, Covill-Cooke et al suggest that Miro proteins are required for short microtubule-independent movements. These findings are interesting and add to the field discussion regarding the regulation of peroxisome mobility and morphology. However, there are several concerns with the experiments and interpretation of the data.
We thank the reviewer for their interest in our manuscript and suggested improvements.

1) Based on the observation of reduced GFP-GTP1 constructs on peroxisomes by confocal microscopy and decreased binding to Pex19, the authors suggest that the first GTPase domain may negatively regulate targeting of Miro1 to peroxisomes via Pex19. This is an over interpretation of the data since there are other more plausible interpretations. The lack of binding may rest on the truncated Miro1 construct itself and not on the GTP1 domain. Miro1 is a tail-anchored targeting protein where its membrane targeting signal is located at the TM and the sequence flanking it. Recently, Okumoto et. al (2017) JCB 217:619-33 have shown that the N-terminal region of the TM plays a role in targeting a Miro1 variant to peroxisomes, suggesting the importance of this region. From the method section, it is not clear how the GFP1 protein was constructed. However, based on the illustration in Figure 2A it appears that the GTP1 construct conside of the first 184 residues of Miro1 at the N-terminus of the TM domain. If this is indeed the case, the construct is likely missing the N-terminus flanking sequence that may be required to bind to Pex19. To test whether the GTP1 domain does regulate Pex19 binding, the authors should increase the length of their TM domain at least from the residue 560 to 618.

We apologise for the omission of the exact residues for the ^{GFP}GTP1 construct in the original manuscript. The construct includes the first GTPase domain (1-184) fused to 562-618 and therefore contains the region upstream of the transmembrane: i.e. all residues after the second GTPase domain (see figure 1D). Importantly, this construct only differs from ^{GFP}GTP2 by the particular GTPase domain in each case (either GTP1 or GTP2), supporting that GTP1 regulates Pex19 binding. This information has now been added to the manuscript.

2) Total fluorescence intensity on peroxisomes is not a good indicator of quantitative measure of protein on peroxisomes (Figure 2C). Instead, the authors should compare subcellular fractionation to demonstrate a difference in the localization of the various constructs to peroxisomes.

We have opted for this approach for a couple of reasons: 1) Fluorescence intensity from confocal imaging is used routinely to measure and compare the presence of a protein of interest between conditions; and 2) Fractionation approaches are population based and are prone to contamination of subcellular compartments. Western blotting of resulting fractions is also only semi-quantitative, as opposed to imaging approaches. To support our findings we have also plotted the data as a ratio of peroxisomal to mitochondrial GFP signal (normalising Miro signal within each cell), accounting for differences in expression. Doing so yields the same result as Figure 1E-F, whereby Miro1 lacking the first GTPase shows a dramatic increase in peroxisomal localisation (Rebuttal figure 3).

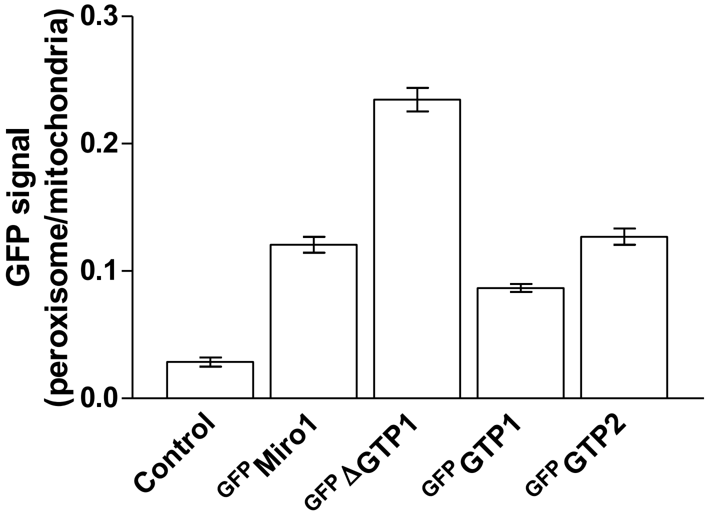

Rebuttal figure 3: Miro intensity on peroxisomes divided by Miro signal on mitochondria.

3) The authors show that the double knock out of Miro1 and Miro2 results in the loss of short-distance movement, but has no effect in the microtubule long distance movement. This result conflicts from the recent work by Okumoto et al. One obvious difference between to two is the use of a double KO MEF versus the RNAi depletion of Miro1. Given that the Okumoto et al manuscript is published, the onus lays on this manuscript to explore the difference. Each system (KO vs RNAi)

has their advantages and disadvantages. The authors should test whether they observe any differences in peroxisome mobility using the RNAi to deplete the cells of Miro1 and Miro2.
We thank the reviewer for their comment and have addressed this concern in two ways: 1) to ascertain the difference of the double KO of Miro1 and Miro2 in our manuscript vs. the single knockout of Miro1 in Okumoto *et al.* (2018) we have studied peroxisomal trafficking in either Miro1 or Miro2 single knockout MEFs. Using these cells we find no difference between WT, Miro1^{KO}, Miro2^{KO} and DKO MEFs in long-range peroxisomal transport (Figure 2A-D). This holds true using two different cell lines for each genotype; 2) As one main advantage of RNAi over constitutive knockout studies is the ability to acutely knockdown a protein of interest we have investigated the effect of acute loss of Miro1 on peroxisomal transport in our system, see final point of reviewer 1. Like the constitutive loss of Miro, acute loss does not affect peroxisomal transport (Figure EV2A-B). These data are further supported by the fact that we observe no alteration in peroxisomal distribution upon loss of Miro1 and Miro2.

3) One novel aspect of this manuscript is the demonstration that Miro2 also targets to peroxisomes. However, the authors do very little to explore the role of Miro2. The authors should explore the role of Miro2 in respect to peroxisome mobility and size by testing whether expressing it in the double KO MEFs can rescue either the short distance movement or rescue its morphology.
We thank the reviewer for this comment and agree that exploring the role of Miro2 in peroxisomal biology would add further novelty. We have therefore added substantial data on Miro2 and the relative contribution of Miro1 and Miro2 to peroxisomes. The following has now been included:

- **We have recently characterised Miro1 single knockout and Miro2 single knockout MEFs (López-Doménech *et al*., 2018), allowing us to study the contribution of either Miro paralogue in peroxisomal trafficking, distribution and size. We explored the effect of Miro1 or Miro2 single knockout on peroxisomal trafficking. Loss of either Miro1 or Miro2 does not affect long-range peroxisomal motility or distribution (Figure 2A-D); however, loss of Miro2 alone did cause a reduction in short-range peroxisomal displacements (Figure 3A-B).**
- **Miro1 and Miro2 appear to be redundant in their role in peroxisomal size. By investigating peroxisomal size in Miro1^{KO} or Miro2^{KO} MEFs (Figure 4A-B) - as well as DKO MEFs rescued with either ^{GFP}Miro1 or ^{GFP}Miro2 (Figure EV4) - the presence of either one of the Miro paralogues maintained peroxisomal size in comparison to WT MEFs. ^{GFP}Miro1 or ^{GFP}Miro2 overexpression in WT MEFs, however, showed that Miro1 may have a stronger effect, as observed by an increase in peroxisomal size upon overexpression of Miro1 but not Miro2 (Figure 4D & F). Therefore, Miro2 can compensate for the loss of Miro1 but Miro1 is likely the driver of the size phenotype.**

4) Could it be possible that the smaller peroxisome size in the double KO cells be due to increased peroxisome division? In Figure 4C, there appears to be more peroxisomes in the DKO cells. The authors should quantify the number of peroxisomes in these cells.
We thank the reviewer for this suggestion. Following quantification of the number of peroxisomes we did indeed find that there are significantly more peroxisomes in the DKO MEFs (Figure 4C). As a result, we have added substantial data regarding the role of Miro in Drp1-dependent fission and have found that Miro negatively regulates the recruitment of Drp1 to both peroxisomes and mitochondria (Figure 5) (please also see bullet-point 3 of the final section of Reviewer 1's comments).

5) Figure 1B and 2C: instead of measuring the area of GFP on peroxisomes, the authors need to measure the GFP on peroxisome compared to total peroxisome area. This is because the total amount or area of peroxisomes can differ between cells, and conditions.
We thank the reviewer for this comment and have now normalised all analysis to peroxisomal area, with no change to the original conclusions.

2nd Editorial Decision 20 May 2019

Thank you for your patience while your revised manuscript was under review. We have now received a complete set of reviews from all referees, which I include below for your information.

As you will see, both referees acknowledge that the manuscript has been improved during the revision, yet both referees still have a number of concerns with the current dataset. Both referees note that the (co-) localization data in the manuscript have not been normalized to total fluorescence and that the observed differences might thus simply be due to different expression levels (GFP-deltaGTP1).

Apart from these concerns, reviewer 1 is not convinced that you made a strong case for the role of Miro1/2 in peroxisomal fission and suggests further experiments to substantiate these findings. Moreover, this reviewer points out that the data on peroxisomal transport contradict earlier findings from the Fujiki and Schrader labs without offering an explanation for this discrepancy.

I have discussed these issues further with both reviewers.
Reviewer 2 agreed with reviewer 1 "[...] that the contradictory findings compared to the two other papers, published more than a year ago, is a problem." This reviewer still recognizes the novelty in your paper "[...] especially if they are correct that Miro proteins are not involved in long range movements. However, the first reviewer is correct, the authors need to better examine and explain the differences between their results."

This reviewer further notes:
"The issue is likely in the difference in the system they used. The main difference between the first two paper (Fujiki and Schrader's laboratories) with this manuscript is that Fujiki and Schrader overexpressed the various isoform of Miro proteins, while this present manuscript used knockout cells. [...] Second, it is possible that there is a compensating system for peroxisome distribution that is masking the function of Miro1/2. Perhaps another Miro isoform or even the ER-peroxisome tethering may be keeping peroxisomes from collapsing to the peri-nuclear area. The point here is that the authors should test their hypothesis using similar reagents used by the other papers. This includes overexpressing Miro1 or Miro2 (tagged with a small tag and not GFP) and examine the distribution of the peroxisomes. Second they should use the siRNA used by Fujiki. According the Fujiki's manuscript their siRNA should knockdown all the isoforms of Miro1.

In short, the authors should investigate and test the findings of the previous work using similar systems. "

Given these opinions, we cannot offer to publish your manuscript in its current form. However, we note that referee 2 is overall supportive of potential publication in EMBO reports and referee 1 agreed with the points raised by this referee during the discussion. We have therefore decided to give you the exceptional possibility of another round of revision to address the remaining concerns. Please also provide a complete point-by-point response to all reviewer concerns.

Revised manuscripts should be submitted within three months of a request for revision; they will otherwise be treated as new submissions. Please contact us if a 3-months time frame is not sufficient for the revisions so that we can discuss the revisions further.

From the editorial side, there are also a few things that we need:

- Your manuscript has currently 5 figures and has been submitted as Scientific report. If the revision results in more than 5 figures, we will publish it as Article, in which case the Results and Discussion section can stay as it is now. If a Scientific Report is submitted, these sections have to be combined. This will help to shorten the manuscript text by eliminating some redundancy that is inevitable when discussing the same experiments twice.

- Figure legends: When displaying bar graphs please ensure to specify the meaning of the bars and error bars (SEM, SD) in the respective figure legends.

- Movies: please follow the nomenclature Movie EVx. Please provide their legend as simple README.txt file and upload one zipped file per movie that contains both, the movie and its respective .txt file.

© European Molecular Biology Organization

I look forward to seeing a revised version of your manuscript when it is ready. Please let me know if you have questions or comments regarding the revision.

****

REFEREE REPORTS

Referee #1:

In this revised manuscript, Covill-Cooke et al. perform studies into the role of the Rho-GTPases Miro1 and Miro2 in peroxisomal morphology and dynamics. In the original submission the authors found that the absence of both proteins (i.e., in a "DKO" strain) led to a decrease in short-distance mobility but not long-distance movement on microtubules, as contrasted a recent report showing effects on long-distance movement. The revised manuscript now shows that the effect on short-distance peroxisomal movement is likely due to ER movement being affected. Furthermore, they show that peroxisomes are smaller and more numerous in the DKO, and find that this correlates with less organellar Drp1-Fis1. They conclude that the role of Miro1 and Miro2 is to negatively regulate peroxisomal fission through Drp1-Fis1 interactions.

Both reviewers asked for more mechanistic information regarding the role of Miro1 and Miro2 on peroxisomal dynamics, and the authors provide good evidence that peroxisomes move through interactions with the ER, and they further show a role of Miro proteins in regulating peroxisomal fission.

I have two main concerns. First, this manuscript leaves us with contradictory findings: two independent groups (PMID: 29222186 and 29364559) now have concluded that Miro1 transports peroxisomes on microtubules. As more than a year has passed since publication of those reports, I don't think it is sufficient for the authors to simply publish contradictory findings. To advance the field they should attempt to reconcile their data with the published results. Is this an issue of cell type, clonal compensation, microscopy methods, or another variable? Publishing the report as is would leave the field confused.

Second, the emphasis (as stated in the title) is now that peroxisomal fission is modulated my Miro1 and Miro2. This is based on smaller and more numerous peroxisomes in the DKO, and a smaller number of cell puncta that are highlighted by Drp1-Fis1 fluorescence. To make the firm conclusion, the authors need to directly compare the rate of fission (i.e., the number of fission events divided by number of peroxisomes over time) in the DKO and wildtype. Live-cell imaging with GFP-SKL labelled peroxisomes would be the obvious way to do this. It shouldn't be too difficult.

There are several other issues that should be addressed:

1) I'm still concerned with the effects of overexpression on targeting and function. Have the authors tried to knock in GFP by gene editing?
2) Figure 1F and 1G: Fig. 1G shows much less expression of GFP-Miro compared with GFP-deltaGTP1. I saw no mention that the GFP signal/peroxisome was normalized for expression. So it seems that the higher localization of deltaGTP1 may simply be due to higher expression! In general, scoring colocalization in the way the authors do it has to be normalized to expression for similar figures as well. By the way, are the black rounded rectangles in Fig. 1D the TM domain or GFP? Both should be shown in the diagram and described in the legend.
3) In Fig. EV1C and 1D, the GFP signal/organelle is determined to be greater than one for some samples. But the colocalization calculation, as described in the methods, is signal divided by the total peroxisomal area. I assumed "1" would indicate that every peroxisomal pixel is positive for GFP, such that "1" is the theoretical maximum. It would be very helpful to associate the signal they report to the % of peroxisomal area to allow better interpretation of the data.
4) The data in Figure 1 shows that GTP2 is as sufficient for mitochondrial targeting as full length. One wonders of the GTP2 domain is important at all for targeting; perhaps only the TM is required?
5) The authors conclude on page 4 an additive effect of exons 19 and 20 based on Fig. EV1C. How can they conclude this as the signal from V4 (containing both exons) is the same as from v2 (containing only exon 19)?
6) The authors conclude that Miro2 preferentially regulates the short-distance migration of

peroxisomes based on Fig. 3B. But the figure shows that the DKO has much lower migration than Miro2KO alone. So both forms seem to be working synergistically.
7) The interpretation to Fig. 3C seems completely off-base: add-back of GFP-v4 should complement the DKO strain by boosting peroxisomal displacement, yet it further impedes movement. This makes no sense to me if the DKO strain is as described.
8) A caveat of the proximity-ligation assay is that mitochondrial and peroxisomal signals are not distinguished from one another. Can't the authors simply co-localize DRP1 or Fis1 to peroxisomes with a peroxisomal marker?
9) Small thing on immunoblots: please state the input amount as 1% of the IP'ed amount, as stated in the authors' rebuttal.
10) Perhaps state that both mouse and human have similar exon splicing, as the authors use the mouse forms while the exon splicing products are described for humans. Are they the same?

Referee #2:

In this revised version, the authors have made substantial improvements to the manuscript with additional experiments and data. The new data on Miro2 adds additional novelty to the study. As before, the experiments are of high quality and the manuscript is much improved. However, there is a concern over their interpretation of the data showing increased del-GTP1 on peroxisomes compared to wild type and the other mutation (Figure 1). Based on the observation that shows increased fluorescent signal from their GFP- del-GTP1 on peroxisomes compared to the full length Miro1 and GFP-GTP1, the authors suggest that the GTP1 domain regulates Miro1 targeting to peroxisomes. However, this data can still be interpreted differently. It can also be interpreted that the region C-terminus of GTP1 (near or on GTP2) is required for its efficient targeting to peroxisomes.

The issue (as discussed in the first review) lies in the use of the total fluorescent intensity of the Miro peroxisome to compare the targeting of the proteins to peroxisomes. This experiment is not an appropriate method to compare protein targeting under the conditions used here. To use total fluorescent intensity to compare proteins levels, there is an assumption that the total amount of proteins expressed in the cell is the same or similar between the different constructs. The problem here is that the protein levels of the different constructs of Miro1 are not expressed at the same levels. This can be seen in Figure 1G where the expression of GFP-Miro is lower than the other constructs. As Miro has two sub-cellular locations (mitochondria and peroxisomes), the ratio of its localization under any given condition will remain constant such that the amount of Miro found on peroxisomes will be directly proportional to its total protein levels, such that a lower expressing protein will have less protein on peroxisomes compared to those with higher expressions. Thus, another interpretation of Figure 1F is that the levels of GFP-del-GTP1 is higher on peroxisomes compared to GFP-Miro due to its higher expression levels. To compare the targeting of these constructs, it is recommended that the authors compare the peroxisome-localized protein to the total level of protein . This can be done by comparing the fluorescent intensity in peroxisomes to that of the entire cell. This reviewer also strongly recommends that the authors validate their assay by performing sub-cellular fractionation to compare the fraction in peroxisomes to the total protein levels in the whole cell lysate. Such an experiment is the gold standard for protein targeting.

2nd Revision - authors' response 7 September 2019

Referee #1

In this revised manuscript, Covill-Cooke et al. perform studies into the role of the Rho-GTPases Miro1 and Miro2 in peroxisomal morphology and dynamics. In the original submission the authors found that the absence of both proteins (i.e., in a "DKO" strain) led to a decrease in short-distance mobility but not long-distance movement on microtubules, as contrasted a recent report showing effects on long-distance movement. The revised manuscript now shows that the effect on short-distance peroxisomal movement is likely due to ER movement being affected. Furthermore, they show that peroxisomes are smaller and more numerous in the DKO, and find that this correlates with less organellar Drp1-Fis1. They conclude that the role of Miro1 and Miro2 is to negatively regulate peroxisomal fission through Drp1-Fis1 interactions.

Both reviewers asked for more mechanistic information regarding the role of Miro1 and Miro2 on peroxisomal dynamics, and the authors provide good evidence that peroxisomes move through interactions with the ER, and they further show a role of Miro proteins in regulating peroxisomal fission.

I have two main concerns. First, this manuscript leaves us with contradictory findings: two independent groups (PMID: 29222186 and 29364559) now have concluded that Miro1 transports peroxisomes on microtubules. As more than a year has passed since publication of those reports, I don't think it is sufficient for the authors to simply publish contradictory findings. To advance the field they should attempt to reconcile their data with the published results. Is this an issue of cell type, clonal compensation, microscopy methods, or another variable? Publishing the report as is would leave the field confused.
We thank the reviewer for their comment and understand the difficulties with contradictory findings. However, we strongly disagree with the implication that in our current study we 'simply publish contradictory findings' as our study provides several important advancements to the field including:

1) **We demonstrate that loss of Miro1 or Miro2 or both Miro proteins does not impact steady-state peroxisome distribution, in stark contrast to what is observed for mitochondria. We think this point is critically important for the field;**
2) **We show for the first time that Miro binding to PEX19 requires the Miro transmembrane domain and that the peroxisomal targeting of Miro is influenced by GTPase domains;**
3) **We demonstrate a role for Miro in short range mitochondrial transport (which, as discussed, may reconcile some of our findings on trafficking with published work);**
4) **We provide several pieces of evidence supporting an important role for Miro proteins in regulating peroxisomal size and fission, highlighting additional roles for Miro beyond trafficking;**
5) **We describe a new PLA-assay for quantifying Drp1 stabilisation on mitochondrial and peroxisomal membranes.**

We would like to emphasise that many of the key conclusions on the importance of Miro for transporting peroxisomes on microtubules (in PMID: 29222186 and 29364559) have been obtained from overexpression studies, which have their own caveats compared to a loss of function approach. In contrast, we have performed either acute or constitutive loss of function experiments which do not support a significant role for Miro in eliciting basal long-range, microtubule-dependent peroxisomal transport, in agreement with the lack of any impact on peroxisome distribution. Further to the evidence provided after the first round of revisions, we have followed the experimental paradigm of Okumoto et al. (2018), namely knocking down Miro1 in HeLa cells, and have attempted to match as close as possible their imaging set-up. We observe no difference in long-range trafficking events between scrambled and Miro1 RNAi (Figure 2H). Importantly, we observe a significant reduction in long-range peroxisomal transport in Pex14 knockdown cells, as a positive control (Figure 2H) [14], highlighting that our analysis can ascertain differences in peroxisomal transport. We believe that the differences in conclusion may in part arise from the types of analysis carried out in the other two studies. For example, the analysis carried out by Castro et al. (2018) shows a shift in the entire cumulative velocity plot, not just what is defined as long-range trafficking (i.e. including short-range peroxisomal displacements which we show require Miro). We have now updated the text of the manuscript to include discussion of these ideas (Page 10 paragraph 2).

Second, the emphasis (as stated in the title) is now that peroxisomal fission is modulated my Miro1 and Miro2. This is based on smaller and more numerous peroxisomes in the DKO, and a smaller number of cell puncta that are highlighted by Drp1-Fis1 fluorescence. To make the firm conclusion, the authors need to directly compare the rate of fission (i.e., the number of fission events divided by number of peroxisomes over time) in the DKO and wildtype. Live-cell imaging with GFP-SKL labelled peroxisomes would be the obvious way to do this. It shouldn't be too difficult.
We respectfully disagree with the reviewer that imaging peroxisomal fission 'shouldn't be too difficult'. Unfortunately, unlike mitochondrial fission, peroxisomal fission events are very difficult to reliably count owing to their small size, vesicular morphology and continuous interaction with one another [1], i.e. it is very challenging to definitively say what is the

disassociation of two interacting peroxisomes and fission of a single peroxisome. We attempted to count fission events in both luminally-targeted GFP (GFP-SKL) and GFP targeted to the peroxisomal membrane. In both cases we were not convinced we could accurately count fission instead of disassociation. Furthermore, we are not aware of papers that count in a quantitative manner peroxisomal fission events in a cell. In fact, in a recent article where mitochondrial fission events are counted, peroxisomal fission is instead quantified through peroxisomal length [11]. We would like to highlight that the approaches we have used to conclude alterations in peroxisomal fission are in complete accordance with those used throughout the peroxisomal fission literature, i.e. size, number and morphology [3–11]. Together with our Drp1 and Fis1 data (including a novel PLA-based assay that we have developed), we feel we make a strong case for a role of Miro in peroxisomal fission.

There are several other issues that should be addressed:

1) I'm still concerned with the effects of overexpression on targeting and function. Have the authors tried to knock in GFP by gene editing?
We agree with the reviewer's point that drawing too many conclusions from artificial overexpression is problematic; this is precisely why our conclusions regarding the function of Miro at peroxisomes are particularly important, as they are predominantly derived from loss-of-function experiments (i.e. are not reliant on overexpression studies). We would also like to point out that all but one of our targeting experiments are performed on a Miro1/2 DKO background and are thus knockout rescue experiments, rather than overexpression. We agree that gene editing would be an interesting approach to further explore Miro1 targeting to peroxisomes but think this is beyond the scope of the current manuscript. Especially, as four previous papers have shown endogenous Miro localised at peroxisomes [12,22–24].
We would like to take this opportunity to highlight that we feel it is somewhat unfair that the issue of overexpression as a potential limitation has been raised. In their comments above, the reviewer points to one of their main concerns being our contradictory findings with Castro et al. (2018) and Okumoto et al. (2018). The Castro et al. paper relies entirely on overexpression (of Miro1 variant 1 artificially targeted to peroxisomes) to draw their key conclusions on the importance of Miro1 for long range transport. Okumoto et al. also use overexpression for many of their key experiments on trafficking and peroxisomal distribution.

2) Figure 1F and 1G: Fig. 1G shows much less expression of GFP-Miro compared with GFP-deltaGTP1. I saw no mention that the GFP signal/peroxisome was normalized for expression. So it seems that the higher localization of deltaGTP1 may simply be due to higher expression! In general, scoring colocalization in the way the authors do it has to be normalized to expression for similar figures as well. By the way, are the black rounded rectangles in Fig. 1D the TM domain or GFP? Both should be shown in the diagram and described in the legend.
We thank the reviewer for this helpful comment (which was also raised by reviewer 2) and have now normalised peroxisomal signal to total fluorescence and included this analysis for all fluorescent quantification. Using this normalisation we find a significant enrichment in the ΔGTP1 construct over full-length Miro1, in agreement with the original manuscript.
We have also updated the schematics in Figure 1D and EV1A, to include the suggested improvements.

3) In Fig. EV1C and 1D, the GFP signal/organelle is determined to be greater than one for some samples. But the colocalization calculation, as described in the methods, is signal divided by the total peroxisomal area. I assumed "1" would indicate that every peroxisomal pixel is positive for GFP, such that "1" is the theoretical maximum. It would be very helpful to associate the signal they report to the % of peroxisomal area to allow better interpretation of the data.
We agree that this analysis is not as clear as possible and have now updated it to be the same as that in Figure 1 (carrying out the suggested normalisation in point 2). Variants 2, 3 and 4 are all enriched over variant 1 on peroxisomes (Figure EV1).

4) The data in Figure 1 shows that GTP2 is as sufficient for mitochondrial targeting as full length. One wonders of the GTP2 domain is important at all for targeting; perhaps only the TM is required?
Following the suggested normalisation of fluorescent data (point 2), we find that ^{GFP}GTP2 is enriched on peroxisomes in comparison to ^{GFP}GTP1 and full-length Miro1. As a result, it is probable that the GTPase2 domain is required for efficient peroxisomal targeting. This could

© European Molecular Biology Organization

be a result of its proximity to the transmembrane and C-terminal amino acids, features that our work and that of Okumoto et al. (2018) show to be important for peroxisomal targeting.

5) The authors conclude on page 4 an additive effect of exons 19 and 20 based on Fig. EV1C. How can they conclude this as the signal from V4 (containing both exons) is the same as from v2 (containing only exon 19)?
Following the renormalisation of fluorescent data to total cell intensity, we find that variant 4 is enriched on peroxisomes in comparison to variant 2 (Figure EV1). Again, we thank the reviewer for their suggestion.

6) The authors conclude that Miro2 preferentially regulates the short-distance migration of peroxisomes based on Fig. 3B. But the figure shows that the DKO has much lower migration than Miro2KO alone. So both forms seem to be working synergistically.
We thank the reviewer for their comment and agree that there might be some compensation from Miro1 in the Miro2^{KO}. As a result, we have softened the language to better represent the data (page 6 of manuscript).

7) The interpretation to Fig. 3C seems completely off-base: add-back of GFP-v4 should complement the DKO strain by boosting peroxisomal displacement, yet it further impedes movement. This makes no sense to me if the DKO strain is as described.
Reviewer 2 suggested testing the function of smaller tagged versions of Miro1 splice variants, to ensure the large GFP tag was not altering the function of Miro1. We have now carried out the assay again and find no change in peroxisomal displacement when reintroducing ^{myc}v4 (Figure 4F).

8) A caveat of the proximity-ligation assay is that mitochondrial and peroxisomal signals are not distinguished from one another. Can't the authors simply co-localize DRP1 or Fis1 to peroxisomes with a peroxisomal marker?
We agree with the reviewer. Indeed, colocalisation of endogenous Drp1 and Fis1 were already included in the previous draft of our manuscript. This analysis showed an increase in the localisation of Drp1 to both mitochondria and peroxisomes in DKO MEFs, as predicted from the PLA assay (Figure 6G-I), and no change in the localisation of Fis1 to either membrane (Figure EV6). Importantly, whole cell levels of Drp1 are not altered in the DKO MEFs, as observed by Western blotting (Figure 6E-F), highlighting that the observed increase in Drp1 recruitment in the DKO MEFs is not an indirect consequence of higher total Drp1 signal. We believe that the PLA further illustrates our findings.

9) Small thing on immunoblots: please state the input amount as 1% of the IP'ed amount, as stated in the authors' rebuttal.
We thank the reviewer for spotting this omission and have now included the amount IP'ed in the figure.

10) Perhaps state that both mouse and human have similar exon splicing, as the authors use the mouse forms while the exon splicing products are described for humans. Are they the same?
We agree that this is a useful inclusion and have therefore updated the manuscript to include this information (page 4 of manuscript).

Referee #2

In this revised version, the authors have made substantial improvements to the manuscript with additional experiments and data. The new data on Miro2 adds additional novelty to the study. As before, the experiments are of high quality and the manuscript is much improved. However, there is a concern over their interpretation of the data showing increased del-GTP1 on peroxisomes compared to wild type and the other mutation (Figure 1). Based on the observation that shows increased fluorescent signal from their GFP- del-GTP1 on peroxisomes compared to the full length Miro1 and GFP-GTP1, the authors suggest that the GTP1 domain regulates Miro1 targeting to peroxisomes. However, this data can still be interpreted differently. It can also be interpreted that the region C-terminus of GTP1 (near or on GTP2) is required for its efficient targeting to peroxisomes.

The issue (as discussed in the first review) lies in the use of the total fluorescent intensity of the Miro peroxisome to compare the targeting of the proteins to peroxisomes. This experiment is not an appropriate method to compare protein targeting under the conditions used here. To use total fluorescent intensity to compare proteins levels, there is an assumption that the total amount of proteins expressed in the cell is the same or similar between the different constructs. The problem here is that the protein levels of the different constructs of Miro1 are not expressed at the same levels. This can be seen in Figure 1G where the expression of GFP-Miro is lower than the other constructs. As Miro has two sub-cellular locations (mitochondria and peroxisomes), the ratio of its localization under any given condition will remain constant such that the amount of Miro found on peroxisomes will be directly proportional to its total protein levels, such that a lower expressing protein will have less protein on peroxisomes compared to those with higher expressions. Thus, another interpretation of Figure 1F is that the levels of GFP-del-GTP1 is higher on peroxisomes compared to GFP-Miro due to its higher expression levels. To compare the targeting of these constructs, it is recommended that the authors compare the peroxisome-localized protein to the total level of protein . This can be done by comparing the fluorescent intensity in peroxisomes to that of the entire cell. This reviewer also strongly recommends that the authors validate their assay by performing sub-cellular fractionation to compare the fraction in peroxisomes to the total protein levels in the whole cell lysate. Such an experiment is the gold standard for protein targeting.

We thank the reviewer for their feedback and for stating that ***'the experiments are of high quality and the manuscript is much improved'*****.**

We agree normalisation to total fluorescence would better control for differences in expression. As a result, we have carried out this normalisation and still observe a dramatic peroxisomal enrichment of $^{GFP}\Delta$GTP1 in comparison to full-length Miro1 (Figure 1F). We now find that ^{GFP}GTP2 is enriched in comparison to full-length Miro1, and therefore agree it is likely that the sequences within the second GTPase domain are important for targeting. We have now included this normalisation for all colocalization analyses in the paper (Figure 1B, 1F and EV1C-D), and updated our conclusions.

Bibliography

1. **Bonekamp NA, Sampaio P, de Abreu FV, Lüers GH, Schrader M (2012) Transient complex interactions of mammalian peroxisomes without exchange of matrix or membrane marker proteins. *Traffic* 13: 960–978.**
2. **Delille HK, Agricola B, Guimaraes SC, Borta H, Lüers GH, Fransen M, Schrader M (2010) Pex11pbeta-mediated growth and division of mammalian peroxisomes follows a maturation pathway. *J Cell Sci* 123: 2750–2762.**
3. **Koch A, Thiemann M, Grabenbauer M, Yoon Y, McNiven MA, Schrader M (2003) Dynamin-like protein 1 is involved in peroxisomal fission. *J Biol Chem* 278: 8597–8605.**
4. **Koch A, Yoon Y, Bonekamp NA, McNiven MA, Schrader M (2005) A role for Fis1 in both mitochondrial and peroxisomal fission in mammalian cells. *Mol Biol Cell* 16: 5077–5086.**
5. **Koch J, Pranjic K, Huber A, Ellinger A, Hartig A, Kragler F, Brocard C (2010) PEX11 family members are membrane elongation factors that coordinate peroxisome proliferation and maintenance. *J Cell Sci* 123: 3389–3400.**
6. **Koch J, Brocard C (2012) PEX11 proteins attract Mff and human Fis1 to coordinate peroxisomal fission. *J Cell Sci* 125: 3813–3826.**
7. **Huber N, Guimaraes S, Schrader M, Suter U, Niemann A (2013) Charcot-Marie-Tooth disease-associated mutants of GDAP1 dissociate its roles in peroxisomal and mitochondrial fission. *EMBO Rep* 14: 545–552.**
8. **Gandre-Babbe S, van der Bliek AM (2008) The novel tail-anchored membrane protein Mff controls mitochondrial and peroxisomal fission in mammalian cells. *Mol Biol Cell* 19: 2402–2412.**
9. **Opaliński Ł, Kiel JAKW, Williams C, Veenhuis M, van der Klei IJ (2011) Membrane curvature during peroxisome fission requires Pex11. *EMBO J* 30: 5–16.**
10. **Williams C, Opalinski L, Landgraf C, Costello J, Schrader M, Krikken AM, Knoops K, Kram AM, Volkmer R, van der Klei IJ (2015) The membrane remodeling protein Pex11p activates the GTPase Dnm1p during peroxisomal fission. *Proc Natl Acad Sci USA* 112: 6377–6382.**

© European Molecular Biology Organization

11. **Kamerkar SC, Kraus F, Sharpe AJ, Pucadyil TJ, Ryan MT (2018) Dynamin-related protein 1 has membrane constricting and severing abilities sufficient for mitochondrial and peroxisomal fission. *Nat Commun* 9: 5239.**
12. **Okumoto K, Ono T, Toyama R, Shimomura A, Nagata A, Fujiki Y (2018) New splicing variants of mitochondrial Rho GTPase-1 (Miro1) transport peroxisomes. *J Cell Biol* 217: 619–633.**
13. **Castro IG, Richards DM, Metz J, Costello JL, Passmore JB, Schrader TA, Gouveia A, Ribeiro D, Schrader M (2018) A role for Mitochondrial Rho GTPase 1 (MIRO1) in motility and membrane dynamics of peroxisomes. *Traffic* 19: 229–242.**
14. **Bharti P, Schliebs W, Schievelbusch T, Neuhaus A, David C, Kock K, Herrmann C, Meyer HE, Wiese S, Warscheid B, et al. (2011) PEX14 is required for microtubule-based peroxisome motility in human cells. *J Cell Sci* 124: 1759–1768.**
15. **Kapitein LC, van Bergeijk P, Lipka J, Keijzer N, Wulf PS, Katrukha EA, Akhmanova A, Hoogenraad CC (2013) Myosin-V opposes microtubule-based cargo transport and drives directional motility on cortical actin. *Curr Biol* 23: 828–834.**
16. **Janssen AFJ, Tas RP, van Bergeijk P, Oost R, Hoogenraad CC, Kapitein LC (2017) Myosin-V Induces Cargo Immobilization and Clustering at the Axon Initial Segment. *Front Cell Neurosci* 11: 260.**
17. **Adrian M, Nijenhuis W, Hoogstraaten RI, Willems J, Kapitein LC (2017) A Phytochrome-Derived Photoswitch for Intracellular Transport. *ACS Synth Biol* 6: 1248–1256.**
18. **Franker MA, Esteves da Silva M, Tas RP, Tortosa E, Cao Y, Frias CP, Janssen AFJ, Wulf PS, Kapitein LC, Hoogenraad CC (2016) Three-Step Model for Polarized Sorting of KIF17 into Dendrites. *Curr Biol* 26: 1705–1712.**
19. **López-Doménech G, Covill-Cooke C, Ivankovic D, Halff EF, Sheehan DF, Norkett R, Birsa N, Kittler JT (2018) Miro proteins coordinate microtubule- and actin-dependent mitochondrial transport and distribution. *EMBO J* 37: 321–336.**
20. **López-Doménech G, Higgs NF, Vaccaro V, Roš H, Arancibia-Cárcamo IL, MacAskill AF, Kittler JT (2016) Loss of Dendritic Complexity Precedes Neurodegeneration in a Mouse Model with Disrupted Mitochondrial Distribution in Mature Dendrites. *Cell Rep* 17: 317–327.**
21. **Guo X, Macleod GT, Wellington A, Hu F, Panchumarthi S, Schoenfield M, Marin L, Charlton MP, Atwood HL, Zinsmaier KE (2005) The GTPase dMiro is required for axonal transport of mitochondria to Drosophila synapses. *Neuron* 47: 379–393.**
22. **Costello JL, Castro IG, Camões F, Schrader TA, McNeall D, Yang J, Giannopoulou E-A, Gomes S, Pogenberg V, Bonekamp NA, et al. (2017) Predicting the targeting of tail-anchored proteins to subcellular compartments in mammalian cells. *J Cell Sci* 130: 1675–1687.**
23. **Wiese S, Gronemeyer T, Ofman R, Kunze M, Grou CP, Almeida JA, Eisenacher M, Stephan C, Hayen H, Schollenberger L, et al. (2007) Proteomics characterization of mouse kidney peroxisomes by tandem mass spectrometry and protein correlation profiling. *Mol Cell Proteomics* 6: 2045–2057.**
24. **Jadot M, Boonen M, Thirion J, Wang N, Xing J, Zhao C, Tannous A, Qian M, Zheng H, Everett JK, et al. (2017) Accounting for protein subcellular localization: A compartmental map of the rat liver proteome. *Mol Cell Proteomics* 16: 194–212.**

3rd Editorial Decision 16 October 2019

Thank you for your patience while we have reviewed your revised manuscript. As you will see from the report below, all concerns have been addressed in this further revised version and also referee 1 is now all positive about its publication in EMBO reports. I am therefore writing with an 'accept in principle' decision, which means that I will be happy to accept your manuscript for publication once a few minor issues/corrections have been addressed, as follows.

- Your manuscript has currently 6 EV figures. Since we can only accommodate up to 5 EV figures, I suggest combining two of these. Please update the figure legends and all callouts in the text accordingly.

© European Molecular Biology Organization

- Our data editors from Wiley have already inspected the Figure legends for completeness and accuracy. Please see all required changes in the attached Word file.

- Finally, EMBO reports papers are accompanied online by A) a short (1-2 sentences) summary of the findings and their significance, B) 2-3 bullet points highlighting key results and C) a synopsis image that is 550x200-400 pixels large (width x height). You can either show a model or key data in the synopsis image. Please note that the size is rather small and that text needs to be readable at the final size. Please send us this information along with the revised manuscript.

Once you have made these minor revisions, please use the following link to submit your corrected manuscript:

https://embor.msubmit.net/cgi-bin/main.plex?el=A3Ij7BJS5A6DxS5J3A9ftdr9UjsdRMhjuxYZDRtOfowY

If all remaining corrections have been attended to, you will then receive an official decision letter from the journal accepting your manuscript for publication in the next available issue of EMBO reports. This letter will also include details of the further steps you need to take for the prompt inclusion of your manuscript in our next available issue.

Thank you for your contribution to EMBO reports.

****

REFEREE REPORT

Referee #1:

I carefully considered the rebuttal of the corresponding author and the revisions. He has appropriately address the problem of conflicting results; I'm satisfied with it. The issue of overexpression also has been dealt with in a satisfactory way. I'm also happy to see more data relating to effect on fission. All minor criticisms has also been addressed. I have no new critical comments and am happy to see this report published without further revision.

3rd Revision - authors' response 8 November 2019

The authors performed all minor editorial changes.

© European Molecular Biology Organization

EMBO PRESS

YOU MUST COMPLETE ALL CELLS WITH A PINK BACKGROUND ↓

PLEASE NOTE THAT THIS CHECKLIST WILL BE PUBLISHED ALONGSIDE YOUR PAPER

Corresponding Author Name: Josef Kittler
Journal Submitted to: EMBO Reports
Manuscript Number: EMBOR-2018-45864V1

Reporting Checklist For Life Sciences Articles (Rev. June 2017)

This checklist is used to ensure good reporting standards and to improve the reproducibility of published results. These guidelines are consistent with the Principles and Guidelines for Reporting Preclinical Research issued by the NIH in 2014. Please follow the journal's authorship guidelines in preparing your manuscript.

A- Figures

1. Data

The data shown in figures should satisfy the following conditions:

- the data were obtained and processed according to the field's best practice and are presented to reflect the results of the experiments in an accurate and unbiased manner.
- figure panels include only data points, measurements or observations that can be compared to each other in a scientifically meaningful way.
- graphs include clearly labeled error bars for independent experiments and sample sizes. Unless justified, error bars should not be shown for technical replicates.
- if n< 5, the individual data points from each experiment should be plotted and any statistical test employed should be justified
- Source Data should be included to report the data underlying graphs. Please follow the guidelines set out in the author ship guidelines on Data Presentation.

2. Captions

Each figure caption should contain the following information, for each panel where they are relevant:

- a specification of the experimental system investigated (eg cell line, species name).
- the assay(s) and method(s) used to carry out the reported observations and measurements
- an explicit mention of the biological and chemical entity(ies) that are being measured.
- an explicit mention of the biological and chemical entity(ies) that are altered/varied/perturbed in a controlled manner.

- the exact sample size (n) for each experimental group/condition, given as a number, not a range;
- a description of the sample collection allowing the reader to understand whether the samples represent technical or biological replicates (including how many animals, litters, cultures, etc.).
- a statement of how many times the experiment shown was independently replicated in the laboratory.
- definitions of statistical methods and measures:
  - common tests, such as t-test (please specify whether paired vs. unpaired), simple χ2 tests, Wilcoxon and Mann-Whitney tests, can be unambiguously identified by name only, but more complex techniques should be described in the methods section;
  - are tests one-sided or two-sided?
  - are there adjustments for multiple comparisons?
  - exact statistical test results, e.g., P values = x but not P values < x;
  - definition of 'center values' as median or average;
  - definition of error bars as s.d. or s.e.m.

Any descriptions too long for the figure legend should be included in the methods section and/or with the source data.

In the pink boxes below, please ensure that the answers to the following questions are reported in the manuscript itself. Every question should be answered. If the question is not relevant to your research, please write NA (non applicable). We encourage you to include a specific subsection in the methods section for statistics, reagents, animal models and human subjects.

USEFUL LINKS FOR COMPLETING THIS FORM

http://www.antibodypedia.com
http://1degreebio.org
http://www.equator-network.org/reporting-guidelines/improving-bioscience-research-repo
http://grants.nih.gov/grants/olaw/olaw.htm
http://www.mrc.ac.uk/Ourresearch/Ethicsresearchguidance/Useofanimals/index.htm
http://ClinicalTrials.gov
http://www.consort-statement.org
http://www.consort-statement.org/checklists/view/32-consort/66-title
http://www.equator-network.org/reporting-guidelines/reporting-recommendations-for-tun
http://datadryad.org
http://figshare.com
http://www.ncbi.nlm.nih.gov/gap
http://www.ebi.ac.uk/ega
http://biomodels.net/
http://biomodels.net/miriam/
http://jjj.biochem.sun.ac.za
http://oba.od.nih.gov/biosecurity/biosecurity_documents.html
http://www.selectagents.gov/

B- Statistics and general methods

Please fill out these boxes ↓ (Do not worry if you cannot see all your text once you press return)

1.a. How was the sample size chosen to ensure adequate power to detect a pre-specified effect size?	No statistical test was used to predetermine size. Sample size was determined by experimental factors. For immunocytochemistry experiments 15-60 cells per condition and experiment were imaged and for western blotting 3 to 5 immunoblots were performed following normal practise in the field. See Figure legends for information on exact numbers used.
1.b. For animal studies, include a statement about sample size estimate even if no statistical methods were used.	No animal studies were used in the manuscript being submitted.
2. Describe inclusion/exclusion criteria if samples or animals were excluded from the analysis. Were the criteria pre-established?	No data points were excluded in any data sets.
3. Were any steps taken to minimize the effects of subjective bias when allocating animals/samples to treatment (e.g. randomization procedure)? If yes, please describe.	N/A
For animal studies, include a statement about randomization even if no randomization was used.	No animal studies were used in the manuscript being submitted.
4.a. Were any steps taken to minimize the effects of subjective bias during group allocation or/and when assessing results (e.g. blinding of the investigator)? If yes please describe.	The raw images for fixed and live imaging experiments were obtained being blind for the channel of interest. The experimenter was blind for genotype when scoring long-range trafficking events. All other analysis was carried out by automated plugin, avoiding experimenter bias at this stage.
4.b. For animal studies, include a statement about blinding even if no blinding was done	No animal studies were used in the manuscript being submitted.
5. For every figure, are statistical tests justified as appropriate?	Yes. In all cases statistical tests are descibed in the appropriate figure legends and the materials and methods section.
Do the data meet the assumptions of the tests (e.g., normal distribution)? Describe any methods used to assess it.	Yes, appropriate tests were chosen based on a criteria of normality, data type (continous, distrete or ordinal) and number of groups. Normality test was carried out using a K-S test against a normal distribution.
Is there an estimate of variation within each group of data?	Yes, an estimate of variation is calculated by an F-test and standard error of the mean for all data-sets.
Is the variance similar between the groups that are being statistically compared?	By F-test, variability in variance was observed between some conditions; however, the statistical test used in all cases was appropriate for the variability within the data sets.

C- Reagents

6. To show that antibodies were profiled for use in the system under study (assay and species), provide a citation, catalog number and/or clone number, supplementary information or reference to an antibody validation profile. e.g., Antibodypedia (see link list at top right), 1DegreeBio (see link list at top right).	Information of antibodies is provided in the material and methods section.
7. Identify the source of cell lines and report if they were recently authenticated (e.g., by STR profiling) and tested for mycoplasma contamination.	MEF cell lines were generated in the lab. We haven't tested mycoplasma contamination.

* for all hyperlinks, please see the table at the top right of the document

D- Animal Models

8. Report species, strain, gender, age of animals and genetic modification status where applicable. Please detail housing and husbandry conditions and the source of animals.	No animals studies were carried out in the manuscript being submitted.
9. For experiments involving live vertebrates, include a statement of compliance with ethical regulations and identify the committee(s) approving the experiments.	No animals studies were carried out in the manuscript being submitted.
10. We recommend consulting the ARRIVE guidelines (see link list at top right) (PLoS Biol. 8(6), e1000412, 2010) to ensure that other relevant aspects of animal studies are adequately reported. See author guidelines, under 'Reporting Guidelines'. See also: NIH (see link list at top right) and MRC (see link list at top right) recommendations. Please confirm compliance.	No animals studies were carried out in the manuscript being submitted.

E- Human Subjects

11. Identify the committee(s) approving the study protocol.	No human subjects were used in the manuscript submitted.
12. Include a statement confirming that informed consent was obtained from all subjects and that the experiments conformed to the principles set out in the WMA Declaration of Helsinki and the Department of Health and Human Services Belmont Report.	No human subjects were used in the manuscript submitted.
13. For publication of patient photos, include a statement confirming that consent to publish was obtained.	No human subjects were used in the manuscript submitted.
14. Report any restrictions on the availability (and/or on the use) of human data or samples.	No human subjects were used in the manuscript submitted.
15. Report the clinical trial registration number (at ClinicalTrials.gov or equivalent), where applicable.	No human subjects were used in the manuscript submitted.
16. For phase II and III randomized controlled trials, please refer to the CONSORT flow diagram (see link list at top right) and submit the CONSORT checklist (see link list at top right) with your submission. See author guidelines, under 'Reporting Guidelines'. Please confirm you have submitted this list.	No human subjects were used in the manuscript submitted.
17. For tumor marker prognostic studies, we recommend that you follow the REMARK reporting guidelines (see link list at top right). See author guidelines, under 'Reporting Guidelines'. Please confirm you have followed these guidelines.	No human subjects were used in the manuscript submitted.

F- Data Accessibility

18: Provide a "Data Availability" section at the end of the Materials & Methods, listing the accession codes for data generated in this study and deposited in a public database (e.g. RNA-Seq data: Gene Expression Omnibus GSE39462, Proteomics data: PRIDE PXD000208 etc.) Please refer to our author guidelines for 'Data Deposition'.<br><br>Data deposition in a public repository is mandatory for:<br>a. Protein, DNA and RNA sequences<br>b. Macromolecular structures<br>c. Crystallographic data for small molecules<br>d. Functional genomics data<br>e. Proteomics and molecular interactions	N/A
19. Deposition is strongly recommended for any datasets that are central and integral to the study; please consider the journal's data policy. If no structured public repository exists for a given data type, we encourage the provision of datasets in the manuscript as a Supplementary Document (see author guidelines under 'Expanded View' or in unstructured repositories such as Dryad (see link list at top right) or Figshare (see link list at top right).	N/A
20. Access to human clinical and genomic datasets should be provided with as few restrictions as possible while respecting ethical obligations to the patients and relevant medical and legal issues. If practically possible and compatible with the individual consent agreement used in the study, such data should be deposited in one of the major public access-controlled repositories such as dbGAP (see link list at top right) or EGA (see link list at top right).	N/A
21. Computational models that are central and integral to a study should be shared without restrictions and provided in a machine-readable form. The relevant accession numbers or links should be provided. When possible, standardized format (SBML, CellML) should be used instead of scripts (e.g. MATLAB). Authors are strongly encouraged to follow the MIRIAM guidelines (see link list at top right) and deposit their model in a public database such as Biomodels (see link list at top right) or JWS Online (see link list at top right). If computer source code is provided with the paper, it should be deposited in a public repository or included in supplementary information.	N/A

G- Dual use research of concern

22. Could your study fall under dual use research restrictions? Please check biosecurity documents (see link list at top right) and list of select agents and toxins (APHIS/CDC) (see link list at top right). According to our biosecurity guidelines, provide a statement only if it could.	Our study does not fall under dual use research restrictions.
